# Changes in Microbial Community Composition Related to Sex and Colon Cancer by Nrf2 Knockout

**DOI:** 10.3389/fcimb.2021.636808

**Published:** 2021-06-23

**Authors:** Chin-Hee Song, Nayoung Kim, Ryoung Hee Nam, Soo In Choi, Jeong Eun Yu, Heewon Nho, Young-Joon Surh

**Affiliations:** ^1^ Department of Internal Medicine, Seoul National University Bundang Hospital, Seongnam, South Korea; ^2^ Department of Internal Medicine and Liver Research Institute, Seoul National University College of Medicine, Seoul, South Korea; ^3^ Tumor Microenvironment Global Core Research Center, College of Pharmacy, Seoul National University, Seoul, South Korea; ^4^ Cancer Research Institute, Seoul National University, Seoul, South Korea

**Keywords:** sex difference, Nrf2 knockout, gut microbiota, AOM/DSS mouse model, colorectal cancer

## Abstract

The frequency of azoxymethane/dextran sulfate sodium (AOM/DSS)-induced carcinogenesis in male mice is higher than that in female mice. Previous studies have reported that 17β-estradiol inhibits tumorigenesis in males by modulating nuclear factor-erythroid 2-related factor 2 (Nrf2). This study aimed to investigate the changes in mouse gut microbiome composition based on sex, AOM/DSS-induced colorectal cancer (CRC), and Nrf2 genotype. The gut microbiome composition was determined by 16S rRNA gene sequencing fecal samples obtained at week 16 post-AOM administration. In terms of sex differences, our results showed that the wild-type (WT) male control mice had higher alpha diversity (i.e. Chao1, Shannon, and Simpson) than the WT female control mice. The linear discriminant analysis effect size (LEfSe) results revealed that the abundances of *Akkermansia muciniphila* and *Lactobacillus murinus* were higher in WT male control mice than in WT female controls. In terms of colon tumorigenesis, the alpha diversity of the male CRC group was lower than that of the male controls in both WT and Nrf2 KO, but did not show such changes in females. Furthermore, the abundance of *A. muciniphila* was higher in male CRC groups than in male controls in both WT and Nrf2 KO. The abundance of *Bacteroides vulgatus* was higher in WT CRC groups than in WT controls in both males and females. However, the abundance of *L. murinus* was lower in WT female CRC and Nrf2 KO male CRC groups than in its controls. The abundance of *A. muciniphila* was not altered by Nrf2 KO. In contrast, the abundances of *L. murinus* and *B. vulgatus* were changed differently by Nrf2 KO depending on sex and CRC. Interestingly, *L. murinus* showed negative correlation with tumor numbers in the whole colon. In addition, *B. vulgatus* showed positive correlation with inflammatory markers (i.e. myeloperoxidase and IL-1β levels), tumor numbers, and high-grade adenoma, especially, developed mucosal and submucosal invasive adenocarcinoma at the distal part of the colon. In conclusion, Nrf2 differentially alters the gut microbiota composition depending on sex and CRC induction.

## Introduction

The gut microbiota maintains homeostasis of host immune and metabolic functions. Dysbiosis is associated with gastrointestinal diseases, cancers, metabolic diseases, and immune disorders (Shanahan, 2013). Previous metagenomic analysis has suggested that patients with colorectal cancer (CRC) are associated with intestinal dysbiosis. The abundance of opportunistic pathogens, such as *Fusobacterium nucleatum*, *Streptococcus bovis*, *S. galloliticus*, *Escherichia coli*, and *Bacteroides fragilis* is enhanced in patients with colorectal adenoma or CRC (Ternes et al., 2020). Additionally, these opportunistic pathogens are considered to be the etiological agents for colorectal carcinogenesis (Sears and Garrett, 2014). A recent study reported that *F. nucleatum* infection promotes metastasis in CRC by activating the autophagy signaling through the upregulation of caspase activation and recruitment domain 3 (CARD3) expression in the CRC mouse model and the HCT116 and SW480 colon cancer cells (Chen et al., 2020). Additionally, *F. nucleatum* promoted chemoresistance to 5-fluorouracil by upregulating the expression of baculoviral IAP repeat-containing 3 (BIRC3) in the CRC cells (Zhang et al., 2019a). However, the role of gut microbiome in the pathogenesis of CRC has not been established.

The incidence of CRC in males is higher than that in females (McCashland et al., 2001) and this sex-specific difference in the incidence of CRC is observed worldwide (Kim et al., 2015). Female sex hormones, especially estrogen, protect against colonic carcinogenesis (Chlebowski et al., 2004; Gierisch et al., 2013). Therefore, menopause is considered a major risk factor for the development of CRC in women. Interestingly, sex hormones also modulate the composition of the gut microbiota (Kim et al., 2020). In particular, estrogen modulates the gut microbiota composition and conversely, the levels of estrogen are also strongly influenced by the gut microbiome (Baker et al., 2017). Furthermore, pathological conditions, such as obesity, diabetes, and cancers adversely affect the cross-talk between estrogen and gut microbiota (Chen and Madak-Erdogan, 2016). Bilateral ovariectomy (OVX), a surgical procedure that mimics post-menopausal status, altered the gut microbial composition of the rodents (Cox-York et al., 2015; Org et al., 2016; Song et al., 2020b). Furthermore, the levels of short-chain fatty acids, which are metabolic signaling molecules produced by the gut microbiota, are markedly downregulated in OVX rats (Cox-York et al., 2015).

Nuclear factor-erythroid 2-related factor 2 (Nrf2) has a dual-role as this can protect the cells from transforming into cancer cells (anticarcinogenic property) and can promote the survival of cancer cells under detrimental conditions (procarcinogenic property) (Lau et al., 2008; Menegon et al., 2016; Son et al., 2019b). Additionally, the aberrant activation or accumulation of Nrf2 is associated with poor clinical outcomes, including cancer development and progression, chemotherapy resistance, and poor survival (Panieri and Saso, 2019). Patients with CRC exhibiting upregulated Nrf2 expression were associated with worse disease-free survival and/or overall survival in the meta-analysis datasets (O’Cathail et al., 2020). Furthermore, the upregulated Nrf2 expression promoted chemotherapy resistance by suppressing the expression of the iron export-related gene SLC40A1 (Wu et al., 2017) and modulating the HER1 signaling pathway (Kankia et al., 2017) in the ovarian cancer cells. The Nrf2 target genes, such as heme oxygenase-1 (HO-1) and NAD(P)H-quinone oxidoreductase-1 (NQO1) are reported to mediate chemotherapy resistance in several cancers, including gastric (Yang et al., 2011) and colon cancers (Schlager and Powis, 1990). Recently, gut bacteria (e.g., *Lactobacillus plantarum* and *Lactobacillus rhamnosus* GG) or microbial metabolites (e.g., urolithin A) were suggested to regulate Nrf2 function (Jones et al., 2015; Singh et al., 2019). However, there is no direct evidence for the Nrf2-mediated regulation of the gut microbiome composition.

Previously, we reported that 17β-estradiol suppresses CRC development in azoxymethane (AOM)/dextran sulfate sodium (DSS)-treated male mice by modulating the Nrf2 signaling pathway (Son et al., 2019b). Additionally, 17β-estradiol altered the gut microbiota composition and consequently modulated the *Firmicutes* to *Bacteroidetes* (F/B) ratio and alpha diversity (Song et al., 2020b). Furthermore, 17β-estradiol strongly inhibited AOM/DSS-induced adenoma/cancer in the distal colon of Nrf2 knockout (KO) male mice but not in that of wild-type (WT) male mice by upregulating estrogen receptor beta (ERβ) expression (Song et al., 2020a). Based on these findings, we hypothesized that the gut microbiome composition is regulated by multiple factors, such as sex, CRC induction, and Nrf2 and that the alteration in gut microbiome composition leads to the development of pathophysiological conditions. To verify this hypothesis, this study focused on the effect of Nrf2 KO on the abundance of sex-specific and CRC-specific taxonomic biomarkers and their correlation with CRC development.

## Materials and Methods

### Mouse Housing Conditions

Nrf2 heterozygous (Nrf2^+/−^) mice with a C57BL/6/129SV background (Chan et al., 1996) were a kind gift from Prof. Y-J Surh (Seoul National University, Korea). WT (Nrf2^+/+^) and homozygous Nrf2 KO (Nrf2^−/−^) mice were obtained by crossing Nrf2 heterozygous (Nrf2^+/−^) mice as previously described (Song et al., 2020a; Song et al., 2021). The Nrf2 KO mice used in the experiment were generated by Chan et al. (1996). Briefly, the basic leucine zipper domain of the Nrf2 gene has been replaced by the lacZ reporter construct by homologous recombination in embryonic stem cells derived from 129SVJ mice. The embryonic stem cells with homologous recombination were injected into the blastocysts of C57BL/6 mice, and chimeric animals with a C57BL/6/129SV mixed background were then bred to produce F1 hybrid mice. And, the heterozygous animals were interbred to produce homozygous knockout animals. The WT and Nrf2 KO mice were housed in cages at 23°C with a 12-h light/dark cycle under specific pathogen-free conditions. All mice were randomly divided based on sex (male and female) and genotype (WT and Nrf2 KO) and housed in the same room in filter-top cages with four mice per cage. The animals were marked so that individual mice could be tracked for the complete duration of the experiments. The experimental procedures were approved by the Institutional Animal Care and Use Committee of the Seoul National University Bundang Hospital (approval number: BA1705-223/043-01). The animal experiments were performed following the Animals in Research: Reporting of *In Vivo* Experiments (ARRIVE) guidelines.

### Study Design and Establishment of the CRC Mouse Model

**Figure 1:**
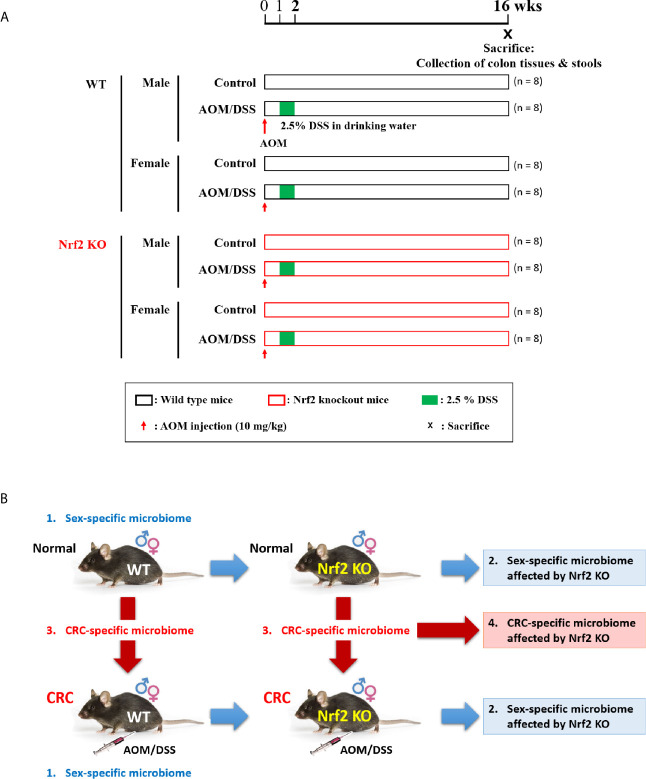
Experimental scheme for evaluating the differential effects of Nrf2 KO on the gut microbial composition depending on sex and CRC induction. **(A)** Experimental design. Male and female WT and Nrf2 KO mice were treated with AOM/DSS to induce colitis-associated CRC. The mice were injected with AOM (10 mg/kg bodyweight) on Day 0. At week 1 post-AOM injection, DSS (2.5% w/v) was supplemented in the drinking water for one week. The fecal samples were collected and the mice were sacrificed at week 16 post-AOM injection (aged 24 weeks). **(B)** Data analysis scheme. Examination of sex-specific changes in the gut microbiome composition by comparing the gut microbiota composition between males and females in the following groups: WT control, WT AOM/DSS-treated, Nrf2 KO control, and Nrf2 KO AOM/DSS-treated groups. Based on the results of this analysis, the effect of Nrf2 KO on the gut microbiome composition was examined. Furthermore, the CRC-specific differences in the gut microbiome composition were analyzed by comparing the gut microbiota composition between control and AOM/DSS-treated animals in the following groups: male WT, female WT, male Nrf2 KO, and female Nrf2 KO groups. Based on this analysis, the effect of Nrf2 KO on the CRC-specific changes in the gut microbiome composition was examined. CRC, colorectal cancer; WT, wild-type; KO, knockout; Nrf2, nuclear factor-erythroid 2-related factor 2; AOM, azoxymethane; DSS, dextran sodium sulfate; ♂, male; ♀, female.

The experimental scheme is presented in **Figure 1A**. The differential effects of Nrf2 KO on the gut microbial composition depending on sex and AOM/DSS treatment were examined. WT mice were divided into the following four groups: male control group (n = 8); male AOM/DSS-treated group (n = 8), female control group; and female AOM/DSS-treated group (n = 8). Similarly, Nrf2 KO mice were divided into the following four groups: male control group (n = 8); male AOM/DSS-treated group (n = 8); female control group (n = 8); and female AOM/DSS-treated group (n = 8). The intestinal microflora alterations in the eight groups of C57BL/6 mice classified based on sex, AOM/DSS treatment, and Nrf2 genotype were comparatively analyzed. The role of Nrf2 in sex-specific and CRC-specific alterations in the microbiome composition (**Figure 1B**) was examined using WT and Nrf2 KO mice. The WT and Nrf2 KO mice were further subdivided based on sex and AOM/DSS treatment. In the AOM/DSS-treated WT and Nrf2 KO groups, only mice with tumors were selected for microbial analysis.

To induce colitis-associated CRC, 5-week-old WT and Nrf2 KO male and female mice were intraperitoneally injected with AOM (10 mg/kg bodyweight; Sigma-Aldrich; A5486), Next, the mice were administered with 2.5% (w/v) DSS (MP Biomedicals, Colitis grade, #160110) through drinking water for 7  days one week after the injection of AOM (Yum et al., 2013) (**Figure 1A**). At week 16 post-AOM injection, fresh fecal samples were collected from all treatment groups (64 samples). All fecal samples were immediately frozen in liquid nitrogen and stored at −80°C until DNA isolation. The animals were euthanized by CO_2_ asphyxiation (**Figure 1A**). The previously reported analysis results of stool samples, which were obtained from the same experimental set, were used to analyze the gut microbiota composition in this study (Song et al., 2020a; Song et al., 2021).

### Stool DNA Extraction and 16S rRNA Sequencing

Genomic DNA was isolated from the frozen fecal samples using the QIAamp DNA stool mini kit (Qiagen, USA). Polymerase chain reaction (PCR) was used to amplify the V3–V4 regions of the 16S rRNA gene with the following primers: 341F, 5’-TCGTCGGCAGCGTCAGATGTGTATAAGAGACAGCCTACGGGNGGCWGCAG-3’ and 805R, 5’-GTCTCGTGGGCTCGGAGATGTGTATAAGAGACAGGACTACHVGGGTATCTAATCC-3’. The amplified products were processed as described previously (Lee et al., 2019). Briefly, the PCR amplification was confirmed using agarose gel electrophoresis. The PCR products were purified using the QIAquick PCR purification kit (Qiagen, USA) and tagged with Illumina index adapters using the Nextera XT index kit (Illumina, USA). Short DNA fragments were removed using a gel/PCR purification kit (Favorgen, Taiwan). PCR amplicons were quantified using the Quant-iT PicoGreen dsDNA assay kit (Thermo Fisher Scientific, USA). DNA samples were pooled (300 ng per sample) and the PCR products purified with a gel/PCR purification kit (Favorgen, Taiwan). The integrity and size of the DNA were analyzed using a Bioanalyzer 2100 (Agilent, USA) with a DNA 7500 chip at ChunLab, Inc. (Seoul, South Korea). Metagenome sequencing was performed using the Illumina MiSeq platform at ChunLab, Inc. (Seoul, South Korea).

### Sequencing Data Processing

The raw reads were first processed based on the read quality. Low quality (< Q25) reads were filtered out using Trimmomatic 0.32 (Bolger et al., 2014). The paired-end sequencing data were merged using PANDAseq (Masella et al., 2012). The primer sequences were then trimmed using an in-house program of the ChunLab, Inc. (Seoul, South Korea) with a similarity cut-off of 0.8. Non-specific amplicons, which do not encode 16S rRNA, were identified using the HMMER program hmmsearch based on the 16S rRNA profiles (Eddy, 2011). The sequences were denoised using DUDE-Seq (Lee et al., 2017), and the non-redundant reads extracted using the UCLUST clustering algorithm (Edgar, 2010). The EzBioCloud database was used for taxonomic assignment with USEARCH (8.1.1861_i86linux32) (Edgar, 2010). Precise pairwise alignment was performed using UCHIME (Edgar et al., 2011). The non-chimeric 16S rRNA database from EzBioCloud was used to detect chimeras for reads with a best hit similarity rate of less than 97%. The sequence data were then clustered using CD-HIT (Fu et al., 2012) and UCLUST (Edgar, 2010).

### Microbiome Analysis

The rarefaction curves of operational taxonomic units (OTUs) were generated using BIOiPLUG (ChunLab, Inc.). To avoid bias in the results, the reads were normalized to 22,146 and then an analysis was performed. Various alpha diversity indices (Chao1, ACE, Jackknife, Shannon, and Simpson) were calculated using OTU information. The Chao1, ACE, and Jackknife are indicators for species richness (total number of species in a sample) through the ratio of singleton or doubleton to the total OTU. The higher value means more species have not been measured, thus, it is considered that species richness is high. The Chao1 index is particularly useful for data sets skewed toward the low-abundance species (Chao, 1984; Hughes et al., 2001). The ACE index is defined as the sum of the probabilities of species observed with fewer than 10 individuals, rather than singletons and doubletons (Chao and Lee, 1992; Hughes et al., 2001). The Jackknife techniques were developed to reduce the bias of a biased estimator, which is the number of species observed in the sample (Burnham and Overton, 1979; Gotelli and Chao, 2013). There is a limited range of sample sizes (near crossing points) where Jackknife estimators are close to the true species richness (Poulin, 1998). The Shannon index is an indicator of species evenness (proportional distribution of the number of each species in a sample) that exhibits values greater than 0 (Shannon, 1948). Higher values indicate higher diversity, and the maximum value is achieved when all species are present in equal numbers. Simpson is an indicator of species evenness that displays the probability that two randomly selected sequences are of the same species (Simpson, 1949). Values range from 0 to 1, and lower values indicate higher diversity. Good’s library coverage estimator is used to measure how much of the real sample’s diversity is covered through sequencing performed (Good, 1953). Sequencing reads used for analysis represents the actual species population of the sample. The value can range from 0 to 100%, with 100% indicating a complete sampling of species, meaning that additional sequencing is unlikely to find any newer species. The differences in the microbiome composition between the treatment groups were visualized using principal coordinate analysis (PCoA). The clustering of the samples was explained based on the principal coordinate values. Additionally, an unweighted pair group method with arithmetic mean (UPGMA) tree was generated using BIOiPLUG (ChunLab, Inc.). The analyses of beta diversity, such as PCoA and UPGMA, were performed using the generalized UniFrac method at the species level. The significance of the separation between the groups was calculated using permutational multivariate analysis of variance (PERMANOVA). A taxonomic bar graph was generated to determine the relative OTU abundance (%) at the phylum and family levels using GraphPad Prism (version 5.01). The abundance of microbes (%) at the levels of phylum, class, order, family, genus, and species are shown in **Supplementary Dataset S1**. The enterotype classification of the gut microbiota at the species level was performed using the R package “clusterSim”. The optimal cluster number was determined by maximizing the value of the Calinski-Harabasz (CH) index, which evaluates cluster effectiveness based on the mean of the sum of squares between clusters and within clusters (Liu et al., 2010).

### Determination of Taxonomic Biomarkers

Based on the relative taxonomic abundance (%), the identification and significance of taxonomic biomarkers were assessed using the linear discriminant analysis (LDA) effect size (LEfSe) method (Segata et al., 2011) at the species level. The general criteria for performing LEfSe were as follows: 1) alpha value of the factorial Kruskal–Wallis *H* test between assigned taxa (when compared with that of the groups) < 0.05; 2) the alpha value for the pairwise Wilcoxon test among the taxonomic members < 0.05; 3) threshold of the logarithmic LDA score for discriminative features < 2.0; and 4) a multi-class analysis set as all-against-all; the LEfSe plot was further simplified as described previously (Song et al., 2020b); 5) overlapping bacteria selection; 6) identifying and classifying the bacterial characteristics based on previous reports as “commensal bacteria,” “opportunistic pathogens,” and “not characterized;” and 7) removal of non-overlapping bacteria from the “not characterized” microbiome. In the LEfSe plot, all commensal bacteria and opportunistic pathogens and only overlapping “not characterized” bacteria were included.

### Evaluation of Clinical Symptoms

Clinical symptoms were evaluated using the disease activity index (DAI), which includes loss of body weight, stool characterization, and hematochezia (Cooper et al., 1993; Park et al., 2015). The DAI was scored by two researchers in a blinded manner. These data have been reported by (Song et al., 2020a; Song et al., 2021).

### Enumeration of Lesions and Histopathology

The colons were dissected longitudinally, and the stool was washed out with phosphate-buffered saline. The length of the colon was measured from the cecum to the rectum using a ruler. Polypoid lesions were independently counted with size measurements using a ruler and macroscopic observation of morphological changes by two researchers in a blinded manner (Cooper et al., 1993; Park et al., 2015). Specifically, colonic segments containing any gross polyps were fixed with phosphate-buffered formalin and embedded in paraffin. The sections were stained with hematoxylin and eosin (H&E). The classification of adenoma and adenocarcinoma, and the specification of the depth of adenocarcinoma invasion into the colonic tissues as mucosal or submucosal invasion was performed by in a blinded manner. These data have been reported by (Song et al., 2020a; Song et al., 2021). In this study, tumor grade was scored as follows: 0, no tumor; 1, low-grade adenoma; 2, high-grade adenoma; 3, mucosal invasive adenocarcinoma; 4, submucosal invasive adenocarcinoma (**Supplementary Dataset S2**).

### Measurement of Inflammatory Cytokines

The levels of myeloperoxidase (MPO) and interleukin (IL)-1β in the colonic tissues were measured using the mouse MPO enzyme-linked immunosorbent assay (ELISA) kit (HK210, Hycult Biotechnology) and mouse IL-1β/IL-1F2 Quantikine ELISA kit (R&D Systems Inc.), respectively. All assays were performed in triplicates.

### Statistical Analysis

All statistical analyses, except those of the pyrosequencing data, were performed using PASW Statistics version 18.0.0 (SPSS Inc., 2009, Chicago, IL, USA). The groups were compared based on the results of the Kruskal–Wallis *H* test. Next, two groups were compared using the Mann–Whitney *U*-test (also known as the Wilcoxon rank-sum test), and the differences were considered significant at *p* < 0.05. For the adjustment of multiple comparisons, the *q*-value of the false positive rate was considered to be less than 5%. Correlation and regression analyses of the microbiome and macroscopic and molecular data were performed using Spearman’s rank correlation coefficient (also known as Spearman’s rho) and linear regression.

### Accession Number

The raw unprocessed gene datasets of 16S rRNA, which were generated in this study, are available in the National Center for Biotechnology Information Sequence Read Archive (Accession number: PRJNA674731; https://www.ncbi.nlm.nih.gov/sra/PRJNA674731).

## Results

This study aimed to analyze the differential effects of Nrf2 KO on the gut microbiota composition depending on sex and CRC induction and to identify the role of Nrf2-mediated bacterial composition alterations in the development of CRC. The design of this study is illustrated in **Figure 1A**. The intestinal microflora alterations in the eight groups of C57BL/6 mice classified based on sex, AOM/DSS treatment, and Nrf2 genotype were comparatively analyzed. The role of Nrf2 in sex-specific and CRC-specific alterations in the microbiome composition (**Figure 1B**) was examined using WT and Nrf2 KO mice. The WT and Nrf2 KO mice were further subdivided based on sex and AOM/DSS treatment. In the AOM/DSS-treated WT and Nrf2 KO groups, only mice with tumors were selected for microbial analysis. To understand the properties of the microbiota found in this study, we analyzed the correlation by taking previously reported results (DAI score, ELISA levels of MPO and IL-1β, and tumor number) from the same set of experiments (Song et al., 2020a; Song et al., 2021). Therefore, some of the data used in this study overlaps with the previous results.

### Differential Effects of Nrf2 KO on the Microbial Composition Depending on Sex and CRC Induction

**Figure 2:**
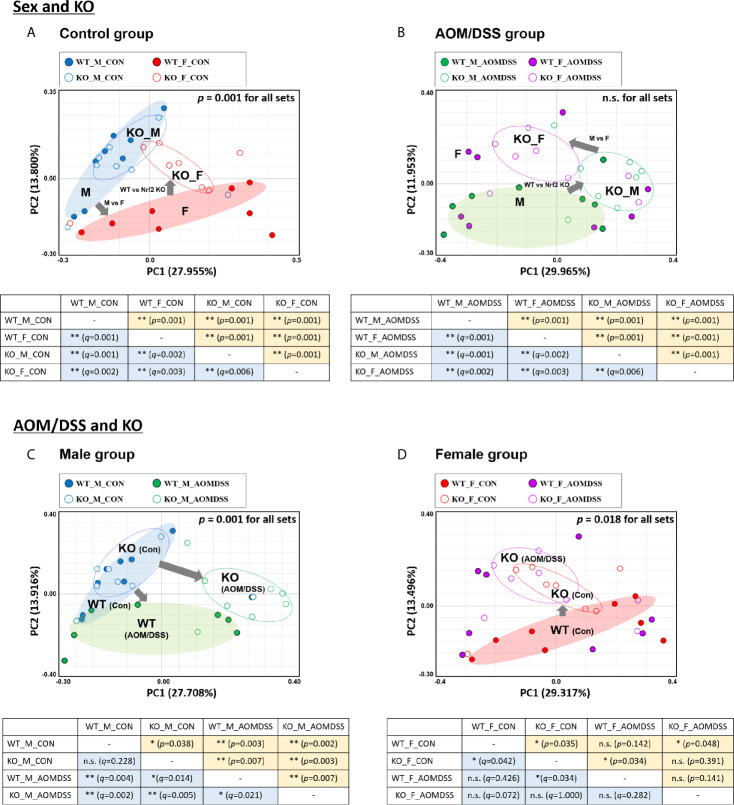
UniFrac-based PCoA of 16S rRNA sequence from 64 fecal samples. Samples derived from control **(A)** and AOM/DSS-treated groups **(B)** with “Sex and KO” criteria, as well as those derived from male **(C)** and female mice **(D)** with “AOM/DSS and KO” criteria, were clustered using the Generalized UniFrac method at the species level. PERMANOVA was performed to examine the dissimilarity of bacterial population structures in **(A–D)**. Below each PCoA plot, the PERMANOVA statistics between the two groups are shown. Significance for *p*- and *q*-values is indicated by asterisks: *, < 0.05; **, < 0.01; n.s. not significance. The clustering of each group is marked with a different color: WT_M_CON, filled blue ellipse; WT_F_CON, filled red ellipse; WT_M_AOM/DSS, filled green ellipse; KO_M_CON, blanked blue ellipse; KO_F_CON, blanked red ellipse; KO_M_AOM/DSS, blanked green ellipse; KO_F_AOM/DSS, blanked purple ellipse. PCoA, principal coordinate analysis; WT, wild-type; KO, Nrf2 knockout; AOM, azoxymethane; DSS, dextran sodium sulfate; M, male; F, female; PERMANOVA, permutational multivariate analysis of variance.

The stool genomic DNA was analyzed by high-throughput 16S rRNA metagenome sequencing. The rarefaction curves of all 64 samples plateaued (**Supplementary Figures S1A, C**). The number of valid reads for each sample is presented in **Supplementary Figures S1B, D**. The gut microbial composition at the species level among different groups was assessed based on the generalized UniFrac distances. The sex-specific differences in the gut microbial composition in the control and AOM/DSS-treated groups were examined. Additionally, the effect of Nrf2 KO on the gut microbial composition in the control and AOM/DSS-treated groups was examined. The results of the PCoA and UPGMA revealed sex-specific alterations in the bacterial composition in both WT and Nrf2 KO control groups (**Figure 2A** and **Supplementary Figures S2A, B, E**). The sex-specific alterations in the gut bacterial composition were observed in the Nrf2 KO AOM/DSS-treated group but not in the WT AOM/DSS-treated group (**Figure 2B** and **Supplementary Figures S2C, D, F**). PERMANOVA for all sets revealed that the beta diversity was significantly different between male and female mice in the control (*p* = 0.001), but not in AOM/DSS-treated groups (**Figures 2A, B** and **Supplementary Figure S2**). Next, the sex-specific differences in the bacterial composition and clustering in the WT and Nrf2 KO AOM/DSS-treated groups were examined. The fecal bacterial composition in the male WT and Nrf2 KO control groups was different from that in the male WT and Nrf2 KO AOM/DSS-treated groups, respectively (**Figure 2C** and **Supplementary Figures S3A, B, E**). Compared with that in the female WT and Nrf2 KO control group, the fecal bacterial composition was different in the female WT and Nrf2 KO AOM/DSS-treated groups (**Figure 2D** and **Supplementary Figures S3C, D, F**). PERMANOVA revealed that the beta diversity was significantly different between male (*p* = 0.001) and female mice (*p* = 0.018) in both control and AOM/DSS-treated groups (**Figures 2C, D** and **Supplementary Figure S3**). These findings indicate that Nrf2 KO altered the fecal bacterial composition in the female WT control group (but not in the WT male control group), as well as in the male and female WT AOM/DSS-treated groups (**Figure 2**). Thus, Nrf2 differentially affected the gut bacterial composition depending on sex and CRC induction.

### Alterations in the Gut Microbiome Diversity Based on Sex, CRC, and Nrf2 Genotype

**Figure 3:**
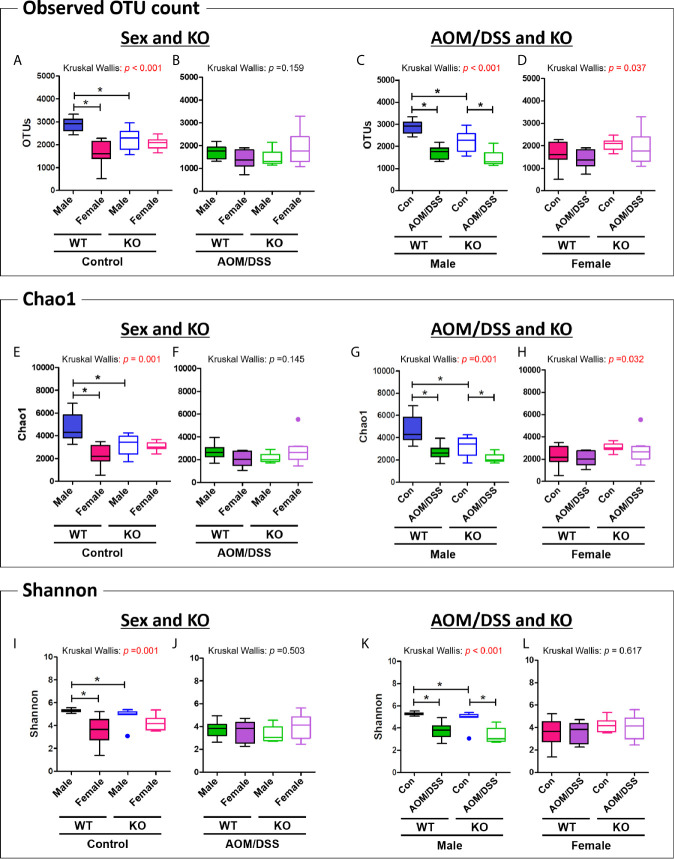
Analysis of species richness and diversity of the gut microbiome. **(A–D)** Observed OTU count, **(E–H)** species richness (Chao1 index), and **(I–L)** alpha diversity (Shannon index) of the microbial community in the groups with “Sex and KO” criteria **(A, B, E, F, I, J)**, as well as with AOM/DSS and KO criteria **(C, D, G, H, K, L)**. Data are expressed as the mean ± SEM. Whiskers show the minimum and maximum values. The *p*-values obtained from the Kruskal–Wallis test is shown in the figure; *p < 0.05 for comparison between two groups (Mann–Whitney U-test). OTU, operational taxonomic unit; WT, wild-type; Nrf2 KO, Nrf2 knockout; Con, control; AOM, azoxymethane; DSS, dextran sodium sulfate; SEM, standard error of mean.

We further analyzed the alpha diversity of the intestinal microbiota based on sex, CRC induction, and Nrf2 genotype. The OTU count in the female WT control group was significantly lower than that in the male WT control group (**Figure 3A** and **Table 1**). However, the sex-specific differences in OTU count were not observed in the Nrf2 KO control groups (**Figure 3A** and **Table 1**). Similarly, the sex-specific differences in the OTU count were not observed in the WT AOM/DSS-treated and Nrf2 KO AOM/DSS-treated groups (**Figure 3B** and **Table 1**). The OTU count in the AOM/DSS-treated group was significantly lower than that in the control group in both male WT and male Nrf2 KO mice (**Figure 3C** and **Table 1**). However, the OTU count did not vary between the AOM/DSS-treated and control groups in both female WT and female Nrf2 KO mice (**Figure 3D** and **Table 1**). Nrf2 KO differentially affected the species richness (Chao1 index) (**Figures 3E–H** and **Table 1**) and alpha diversity (Shannon index) (**Figures 3I–L** and **Table 1**) of the gut microbiota depending on sex and AOM/DSS treatment. Interestingly, the gut microbial diversity indices, including OTU count, Chao1 index, and Shannon index, in the male Nrf2 KO control group were significantly lower than those in the male WT control group (**Figure 3** and **Table 1**). These results indicate that Nrf2 KO differentially altered the intestinal microbial diversity depending on sex and CRC induction.

**Table 1:** Alpha diversity of microbiota from fecal contents.

Group	No. of OTUs	Good’s library coverage (%)	Alpha-diversity				
			ACE	Chao1	Jackknife	Shannon	Simpson
WT_Male_Con (n=8)	2881	93.34	5924.27	4692.82	5605.38	5.29	0.04
WT_Male_AOM/DSS (n=8)	1720	96.07	3414.55	2681.89	3124.66	3.75	0.17
WT_Female_Con (n=8)	1628	96.75	2566.63	2247.64	2505.48	3.59	0.21
WT_Female_AOM/DSS (n=8)	1378	97.08	2524.45	2053.97	2409.87	3.61	0.18
KO_Male_Con (n=8)	2228	95.56	3635.84	3179.50	3653.46	4.85	0.06
KO_Male_AOM/DSS (n=8)	1439	96.87	2429.17	2137.42	2410.95	3.32	0.20
KO_Female_Con (n=8)	2050	95.38	3558.68	3063.25	3514.81	4.20	0.10
KO_Female_AOM/DSS (n=8)	1924	95.93	3244.33	2816.79	3225.92	4.07	0.14
***p-*value for sex differences**							
WT_M_Con *vs* WT_F_Con	<0.001*	0.001*	0.001*	0.001*	0.001*	0.001*	0.007*
KO_M_Con *vs* KO_F_Con	0.382	0.721	0.574	0.645	0.721	0.065	0.065
WT_M_AOM/DSS *vs* WT_F_AOM/DSS	0.105	0.065	0.130	0.083	0.195	0.878	0.878
KO_M_AOM/DSS *vs* KO_F_AOM/DSS	0.234	0.195	0.130	0.195	0.130	0.279	0.195
***p-*value for CRC development**							
WT_M_Con *vs* WT_M_AOM/DSS	<0.001*	0.001*	0.005*	0.002*	0.003*	< 0.001*	0.001*
WT_F_Con *vs* WT_F_AOM/DSS	0.279	0.645	0.798	0.645	0.878	0.798	0.959
KO_M_Con *vs* KO_M_AOM/DSS	0.003*	0.038*	0.028*	0.028*	0.038*	0.001*	0.003*
KO_F_Con *vs* KO_F_AOM/DSS	0.442	0.161	0.195	0.234	0.161	1.000	0.798
***p-*value for Nrf2 KO condition**							
WT_M_Con *vs* KO_M_Con	0.007*	0.028*	0.028*	0.028*	0.028*	0.028*	0.645*
WT_F_Con *vs* KO_F_Con	0.105	0.049*	0.065	0.065	0.049*	0.279	0.234
WT_M_AOM/DSS *vs* KO_M_AOM/DSS	0.083	0.065	0.049*	0.083	0.083	0.234	0.328
WT_F_AOM/DSS *vs* KO_F_AOM/DSS	0.130	0.195	0.328	0.161	0.382	0.382	0.505

Data are presented as the medians. p-value < 0.05 was considered to be significant and was presented as boldface and asterisk. Mann–Whitney U-test for comparison difference between independent two groups was performed. OTU, operational taxonomic unit; WT, wild-type; KO, Nrf2 knockout; Con, control; AOM, azoxymethane; DSS, dextran sulfate sodium salt.

### Changes in Microbial Taxonomic Composition Based on Sex, CRC, and Nrf2 KO at the Phylum and Family Levels

**Figure 4:**
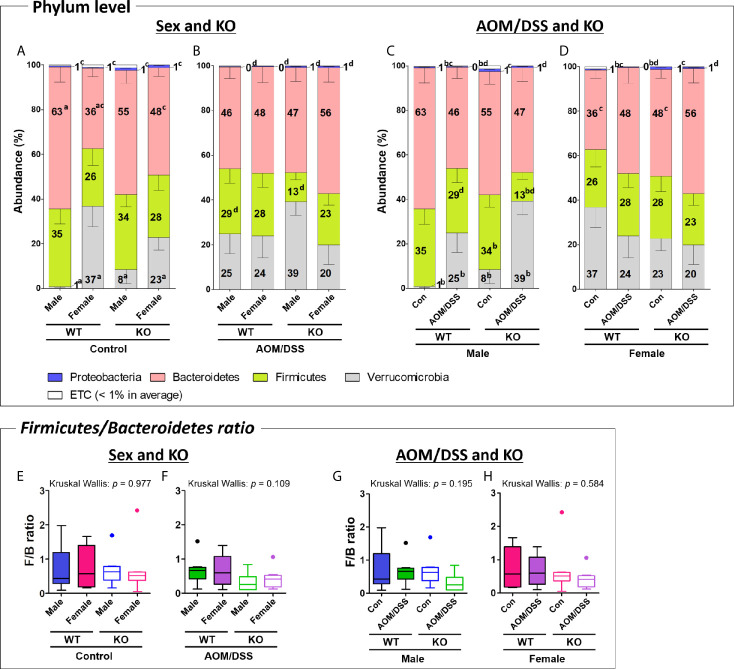
Gut microbiota composition at the phylum level. **(A–D)** Taxonomic composition of fecal samples obtained from the groups with “Sex and KO” criteria **(A, B)**, as well as with “AOM/DSS and KO” criteria **(C, D)**. The abundance indicates the percentage of each phylum in total microorganisms. Stacked bars indicate mean ± SEM. Mann–Whitney U-test was used to analyze the difference between two independent groups in **(A–D)**. ^a^ *p* < 0.05, males *vs*. females; ^b^ *p* < 0.05, controls *vs*. AOM/DSS-treated groups; ^c^ *p* < 0.05, WT controls *vs*. Nrf2 KO controls; ^d^ *p* < 0.05, WT AOM/DSS-treated *vs*. Nrf2 KO AOM/DSS-treated. **(E–H)** *Firmicutes/Bacteroidetes* ratio calculated by dividing the abundance of *Firmicutes* with that of *Bacteroidetes* in the groups with “Sex and KO” criteria, as well as with “AOM/DSS and KO” criteria. Data are expressed as the mean ± SEM. Whiskers show the minimum and maximum values. The *p*-values calculated from the Kruskal–Wallis test is shown in the figure. WT, wild-type; Nrf2 KO, Nrf2 knockout; Con, control; AOM, azoxymethane; DSS, dextran sodium sulfate; SEM, standard error of mean.

The gut microbiota composition at the phylum and family levels varied depending on sex and CRC induction (**Figure 4** and **Supplementary Figure S4-S5**). At the phylum level, the abundance of only *Bacteroidetes* and *Verrucomicrobia* varied between male and female mice. The abundance of *Bacteroidetes* in the female WT control group (average 36%) was significantly lower than that in the male WT control group (63%) (**Figures 4A** and **Supplementary Figure S4E**). However, the sex-specific changes in the abundance of *Bacteroidetes* were not observed in the Nrf2 KO and/or AOM/DSS-treated groups (**Figures 4A, B**). The abundance of *Verrucomicrobia* in the female WT (37%) and Nrf2 KO (23%) control groups significantly higher than that in the male WT (1%) and Nrf2 KO (8%) control groups (**Figure 4A**). However, the sex-specific changes in the abundance of *Verrucomicrobia* were not observed in the AOM/DSS-treated group (**Figure 4B**). At the family level, sex-specific changes in the abundance of *Rikenellaceae*, *Prevotellaceae*, *Odoribacteraceae*, *Muribaculaceae* (phylum: *Bacteroidetes*), *Ruminococcaceae*, *Lactobacillaceae* (phylum: *Firmicutes*), and *Akkermansiaceae* (phylum: *Verrucomicrobia*) were observed in all groups (**Supplementary Figures S5A, B**).

At the phylum level, AOM/DSS treatment significantly altered the abundance of *Firmicutes*, *Verrucomicrobia*, and *Proteobacteria*. The abundance of *Firmicutes* in the male Nrf2 KO AOM/DSS-treated group (13%) was lower than that in the male Nrf2 KO control group (34%). However, the sex-specific changes in the abundance of *Firmicutes* were not observed in the male WT group (**Figures 4C** and **Supplementary Figure S4C**). Additionally, the abundance of *Proteobacteria* in the male and female AOM/DSS-treated WT groups (0.3% in both males and females) was significantly lower than that in the male and female control WT groups (0.6% and 0.5% in males and females, respectively). However, the sex-specific changes in the abundance of *Proteobacteria* were not observed in the control and AOM/DSS-treated Nrf2 KO groups (**Figures 4C, D**). In contrast, the abundance of *Verrucomicrobia* in the male WT and Nrf2 KO AOM/DSS-treated groups (25% and 39% in WT and Nrf2 KO, respectively) was higher than that in the male WT and Nrf2 KO control groups (1% and 8% in WT and Nrf2 KO, respectively) (**Figure 4C**). However, the abundance of *Verrucomicrobia* was not affected in the female WT and Nrf2 KO AOM/DSS-treated groups (**Figure 4D**). At the family level, AOM/DSS treatment altered the abundance of *Rikenellaceae*, *Muribaculaceae*, and *Bacteroidaceae* (phylum *Bacteroidetes*), *Lactobacillaceae*, and *Lachnospiraceae* (phylum *Firmicutes*), and *Akkermansiaceae* (phylum *Verrucomicrobia*) in all treatment groups (**Supplementary Figures S5C, D**).

Nrf2 KO altered the intestinal microbial composition at the phylum level, especially the abundance of *Firmicutes*, *Bacteroidetes*, and *Proteobacteria*. The abundance of *Firmicutes* in the male Nrf2 KO AOM/DSS-treated group (13%) was lower than that in the male WT AOM/DSS-treated (29%) group (**Figure 4B, C** and **Supplementary Figures S4B, C**). Compared with that in the female WT control group (36%), the abundance of *Bacteroidetes* was higher in the female Nrf2 KO control group (48%) (**Figures 4A, D** and **Supplementary Figures S4E, H**). Interestingly, the abundance of *Proteobacteria* in all Nrf2 KO (1.2%, 0.7%, 1.1%, and 0.7% in the male Nrf2 KO control, male Nrf2 KO AOM/DSS-treated, female Nrf2 KO control, and female Nrf2 KO AOM/DSS-treated groups, respectively) groups was significantly higher than that in all WT (0.6%, 0.3%, 0.5%, and 0.3% in the male WT control, male WT AOM/DSS-treated, female WT control, and female AOM/DSS-treated groups, respectively) groups (**Figures 4A–D**). At the family level, Nrf2 KO altered the abundance of *Rikenellaceae*, *Muribaculaceae*, *Bacteroidaceae* (phylum: *Bacteroidetes*), *Lactobacillaceae*, and *Lachnospiraceae* (phylum: *Firmicutes*) in all groups (**Supplementary Figure S5**). Interestingly, the abundance of *Akkermansiaceae* (phylum *Verrucomicrobia*) was not affected by Nrf2 KO.

We further analyzed the ratio of F/B, which are the two major phyla of the domain bacteria. The F/B ratio did not significantly differ among the different groups (**Figures 4E–H**) even though the abundance of *Firmicutes* and *Bacteroidetes* varied depending on sex, AOM/DSS treatment, or Nrf2 genotype (**Supplementary Figure S4**).

### Identification of Sex-Specific and CRC-Specific Taxonomic Biomarkers in the Gut Microbiota

**Figure 5:**
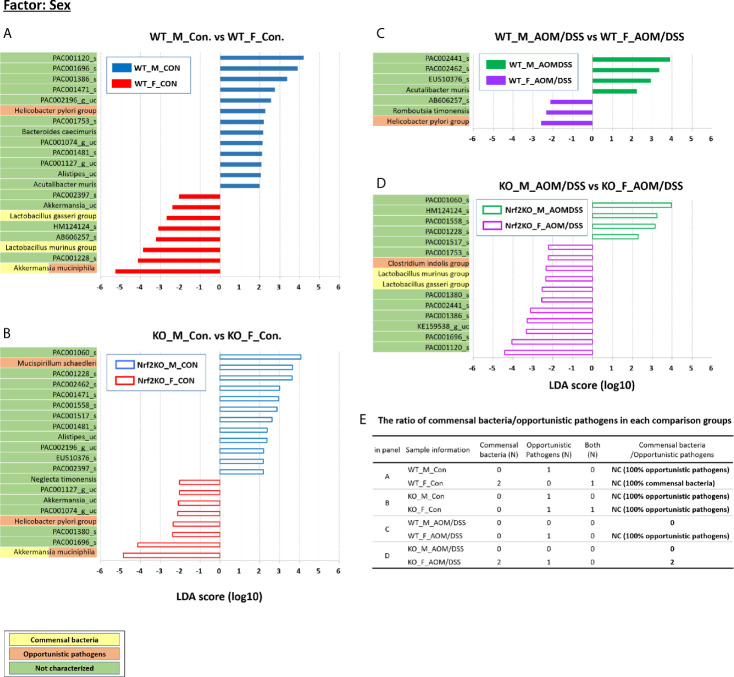
Sex-specific alterations in the abundance of the gut microbiota composition examined using LEfSe. Bar plots of the LEfSe results, which were obtained based on the following criteria: 1) the alpha value for the factorial Kruskal–Wallis *H* test between assigned taxa when compared with that of the groups < 0.05; 2) the alpha value for the pairwise Wilcoxon test among the taxonomic compositions < 0.05; 3) the threshold of the logarithmic LDA score for discriminative features < 2.0; 4) a multi-class analysis set as all-against-all; the LEfSe plot was further simplified; 5) overlapping bacteria selection; 6) identification and classification of the bacterial characteristics based on previous reports as “commensal bacteria,” “opportunistic pathogens,” and “not characterized;” and 7) removal of non-overlapping bacteria only at “not characterized” microbiome. In the LEfSe plot, all commensal bacteria and opportunistic pathogens and only overlapped “not characterized” bacteria are included. Bar plots at the species level with significant differences in abundance based on LEfSe. The color bars and lines show the LDA scores of species that enriched in indicated condition; **(A)** blue bar (WT male controls), **(A)** red bar (WT female controls), **(B)** blue line (Nrf2 KO male controls), **(B)** red line (Nrf2 KO female controls), **(C)** green bar (WT male AOM/DSS-induced CRC), **(C)** purple bar (WT female AOM/DSS-induced CRC), **(D)** green line (Nrf2 KO male AOM/DSS-induced CRC), and **(D)** purple line (Nrf2 KO female AOM/DSS-induced CRC). Each color on the species name indicates the characteristics of each genus: yellow for commensal bacteria, orange for opportunistic pathogens, and green for not characterized bacteria. The *p*-values were determined using the non-parametric factorial Kruskal–Wallis sum-rank test. **(E)** The ratio of commensal bacteria to opportunistic pathogens based on the LEfSe results in each comparison group. LDA, linear discriminant analysis; LEfSe, LDA effect size; CRC, colorectal cancer; WT, wild-type; KO, Nrf2 knockout; Con, control; AOM, azoxymethane; DSS, dextran sodium sulfate; M, male; F, female; N, number; NC, not calculated.

**Figure 6:**
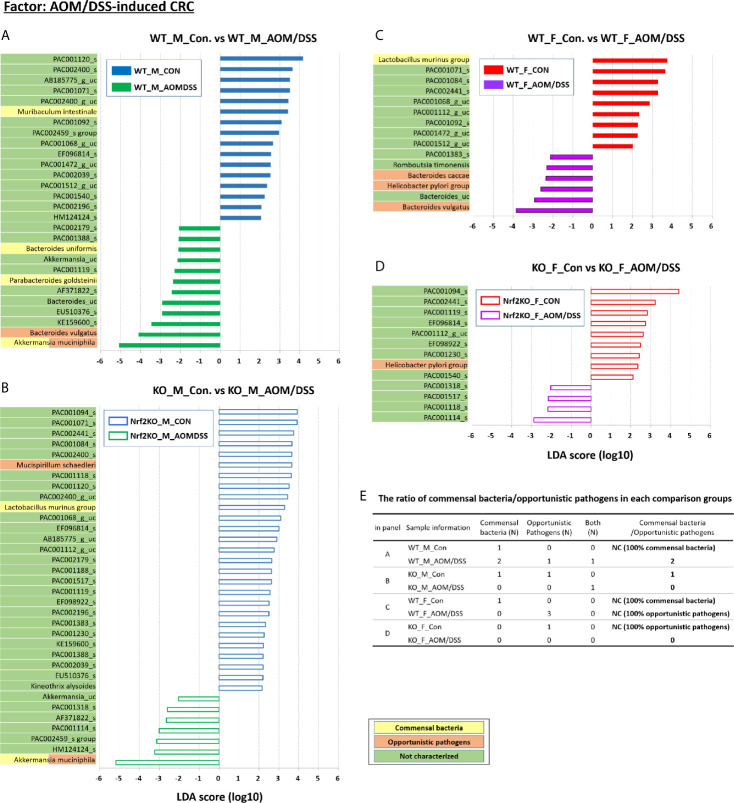
CRC-specific alterations in the abundance of gut microbiota examined using LEfSe. Bar plots of the LEfSe results, which were generated based on the criteria mentioned in the legend of Figure 5. Bar plots at the species level with significant differences in abundance based on LEfSe. The color bars and lines show the LDA scores of species that were enriched in the indicated condition; **(A)** blue bar (WT male controls), **(A)** green bar (WT male AOM/DSS-induced CRC), **(B)** blue line (Nrf2 KO male controls), **(B)** green line (Nrf2 KO male AOM/DSS-induced CRC), **(C)** red bar (WT female controls), **(C)** purple bar (WT female AOM/DSS-induced CRC), **(D)** red line (Nrf2 KO female controls), and **(D)** purple line (Nrf2 KO female AOM/DSS-induced CRC). Each color on the species name indicates the characteristics of each genus: yellow for commensal bacteria, orange for opportunistic pathogens, and green for not characterized bacteria. The *p*-values were calculated using the non-parametric factorial Kruskal–Wallis sum-rank test. **(E)** The ratio of commensal bacteria to opportunistic pathogens based on the LEfSe results in each comparison group. CRC, colorectal cancer; LDA, linear discriminant analysis; LEfSe, LDA effect size; WT, wild-type; KO, Nrf2 knockout; Con, control; AOM, azoxymethane; DSS, dextran sodium sulfate; M, male; F, female; N, number; NC, not calculated.

**Figure 7:**
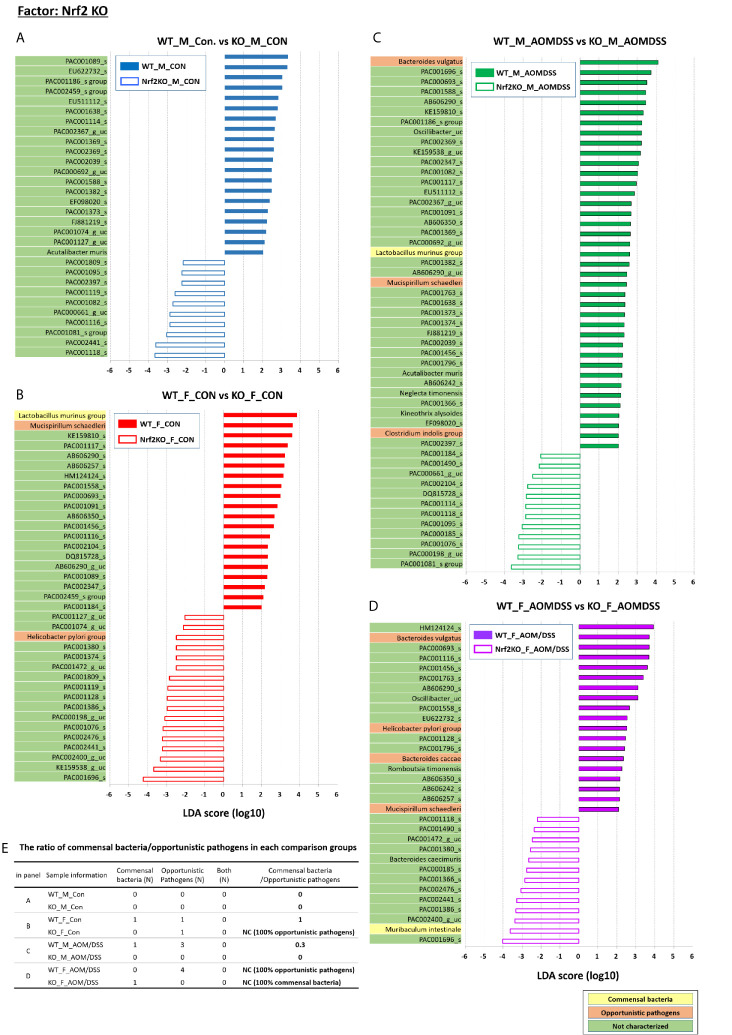
Nrf2 KO-specific alterations in the abundance of gut microbiota examined using LEfSe. Bar plots of the LEfSe results, which were generated based on the criteria mentioned in the legend of **Figure 5**. Bar plots at the species level with significant differences in abundance based on the LEfSe results. The color bars and lines show the LDA scores of species that are enriched in the indicated condition; **(A)** blue bar (WT male controls), **(A)** blue line (Nrf2 KO male controls), **(B)** red bar (WT female controls), **(B)** red line (Nrf2 KO female controls), **(C)** green bar (WT male AOM/DSS-induced CRC), **(C)** green line (Nrf2 KO male AOM/DSS-induced CRC), **(D)** purple bar (WT female AOM/DSS-induced CRC), and **(D)** purple line (Nrf2 KO female AOM/DSS-induced CRC). Each color on the species name indicates the characteristics of each genus: yellow for commensal bacteria, orange for opportunistic pathogens, and green for not characterized bacteria. The *p*-values were determined using the non-parametric factorial Kruskal–Wallis sum-rank test. **(E)** The ratio of commensal bacteria to opportunistic pathogens based on the LEfSe results in each comparison group. CRC, colorectal cancer; LDA, linear discriminant analysis; LEfSe, LDA effect size; WT, wild-type; KO, Nrf2 knockout; Con, control; AOM, azoxymethane; DSS, dextran sodium sulfate; M, male; F, female; N, number; NC, not calculated.

LEfSe of gut microbiota at the species level was performed to identify sex-specific, CRC-specific, and Nrf2 KO-specific taxonomic biomarkers. The abundance of 11 gut bacteria, which were functionally known as commensal bacteria or opportunistic pathogens, significantly changed in this study (**Figures 5**–**7**).

Additionally, LEfSe revealed alterations in the abundance of six sex-specific bacteria (two commensal bacteria, *Lactobacillus gasseri* and *Lactobacillus murinus*; three opportunistic pathogens, *Helicobacter pylori*, *Mucispirillum schaedleri*, and *Clostridium indolis*; one dual-role bacterium, *Akkermansia muciniphila*) (**Figure 5**), nine CRC-specific bacteria (four commensal bacteria, *Muribaculum intestinale*, *Bacteroides uniformis*, *Parabacteroides goldsteinii*, *L. murinus*; four opportunistic pathogens, *Bacteroides vulgatus*, *M. schaedleri*, *Bacteroides caccae*, and *H. pylori*; one dual-role bacterium, *A. muciniphila*) (**Figure 6**), and seven Nrf2 KO-specific bacteria (two commensal bacteria, *L. murinus* and *M. intestinale*; five opportunistic pathogens, *M. schaedleri*, *H. pylori*, *B. vulgatus*, *C. indolis*, and *B. caccae*) (**Figure 7**). The analysis of gut bacteria with an abundance of more than 1% revealed two sex-specific bacteria (*A. muciniphila* and *L. murinus*), four CRC-specific bacteria (*A. muciniphila*, *B. vulgatus*, *L. murinus*, and *M. intestinale*), and three Nrf2 KO-specific bacteria (*B. vulgatus*, *L. murinus*, and *M. intestinale*).

The abundance of *A. muciniphila* in the female WT and Nrf2 KO control groups (36.77% and 22.77%, respectively) was higher than that in the male WT and Nrf2 KO control group (0.84% and 8.35%, respectively) (*p* = 0.001 for male WT control *vs*. female WT control and *p* = 0.046 for male Nrf2 KO control *vs*. female Nrf2 KO control). However, the sex-specific changes in the abundance of *A. muciniphila* were not observed in the WT and Nrf2 KO AOM/DSS-treated groups (**Figures 5A–D**). The abundance of *L. murinus* in the female WT control group was higher than that in the male WT control group (*p* = 0.003) (**Figure 5A**). However, the sex-specific changes in the abundance of *L. murinus* were not observed in the WT AOM/DSS-treated and Nrf2 KO control groups (**Figures 5B, C**). The female Nrf2 KO AOM/DSS-treated group exhibited a higher abundance of *L. murinus* than the male Nrf2 KO AOM/DSS-treated group (*p* = 0.049) (**Figure 5D**). The ratio of commensal bacteria to opportunistic pathogens in the female WT control and female Nrf2 KO AOM/DSS-treated groups was higher than that in the male WT control and male Nrf2 KO AOM/DSS-treated groups, respectively (**Figure 5E**).

At week 16 post-AOM administration, the abundance of *A. muciniphila* in the male WT AOM/DSS-treated and Nrf2 KO AOM/DSS-treated groups was higher than that in the male WT control and male Nrf2 control groups (0.84%, 24.84%, 8.35%, and 39.13% in the male WT control, male WT AOM/DSS-treated, male Nrf2 KO control, and male Nrf2 KO AOM/DSS-treated groups, respectively) (*p* = 0.036 for male WT control *vs*. WT AOM/DSS-treated and *p* = 0.013 for male Nrf2 KO control *vs*. male Nrf2 KO AOM/DSS-treated) (**Figures 6A, B**). Interestingly, the abundance of *A. muciniphila* was not altered in the female WT and female Nrf2 KO AOM/DSS-treated groups (**Figures 6C, D**). The abundance of *B. vulgatus* in the male and female WT AOM/DSS-treated groups was higher than that in the male and female WT control groups (0.60%, 3.15%, 0.33%, and 1.74% in the male WT control, male WT AOM/DSS-treated, female WT control, and female WT AOM/DSS-treated groups, respectively) (*p* = 0.006 for male WT control *vs*. male WT AOM/DSS-treated and *p* = 0.002 for female WT control *vs*. female AOM/DSS-treated) (**Figures 6A, C**). However, the abundance of *B. vulgatus* was not affected in either male or female mice of the Nrf2 KO AOM/DSS-treated groups (**Figures 6B, D**). The abundance of the two selected commensal bacterial species, which are *L. murinus* and *M. intestinale*, in the AOM/DSS-treated groups was lower than that in the control groups. Compared with that in the male WT and male Nrf2 KO control groups, the abundance of *L. murinus* was lower in the female WT and female Nrf2 KO AOM/DSS-treated groups (*p* = 0.021 for female WT control *vs*. female AOM/DSS-treated and *p* = 0.005 for male Nrf2 KO control *vs*. male Nrf2 KO AOM/DSS-treated) (**Figures 6B, C**). However, the sex-specific changes in the abundance of *L. murinus* were not observed in the male WT and female Nrf2 KO AOM/DSS-treated groups (**Figure 6A, D**). The abundance of *M. intestinale* in the male WT AOM/DSS-treated group was lower than that in the male WT control group (*p* = 0.036 for male WT control *vs*. male WT AOM/DSS-treated) (**Figure 6A**). Additionally, the abundance of *M. intestinale* was not affected in other groups (**Figures 6B–D**). The ratio of commensal bacteria to opportunistic pathogens in the female WT AOM/DSS-treated group was lower than that in the female WT control group (**Figure 6E**). Interestingly, the abundance of both commensal bacteria and opportunistic pathogens in both male and female mice of the AOM/DSS-treated Nrf2 KO groups was not upregulated when compared with that in the male and female mice of the Nrf2 KO control groups (**Figure 6E**).

The abundance of *B. vulgatus* in the male Nrf2 KO AOM/DSS-treated group was lower than that in the WT male AOM/DSS-treated group (*p* = 0. 005 for male WT AOM/DSS-treated *vs*. male Nrf2 KO AOM/DSS-treated and *p* = 0. 027 for female WT AOM/DSS-treated *vs*. female Nrf2 KO AOM/DSS-treated) (**Figures 7C, D**). However, the abundance of *B. vulgatus* was not affected in the control groups (**Figures 7A, B**). The abundance of *L. murinus* in the Nrf2 KO group was lower than that in the WT group (*p* = 0. 015 for male WT AOM/DSS-treated vs. male Nrf2 KO AOM/DSS-treated and *p* = 0. 001 for female WT control vs. female Nrf2 KO control) (**Figures 7B, C**). However, the abundance of *M. intestinale* in the female Nrf2 KO AOM/DSS-treated group was higher than that in the female WT AOM/DSS-treated group (*p* = 0. 021) (**Figure 7D**). Additionally, there was no difference in the abundance of *M. intestinale* between the other Nrf2 KO and WT groups. The ratio of commensal bacteria to opportunistic pathogens in the female Nrf2 KO control group was lower than that in the female WT control group. However, there was no difference in this ratio between the male Nrf2 KO and WT control groups (**Figure 7E**). Furthermore, the ratio of commensal bacteria to opportunistic pathogens in the female Nrf2 KO AOM/DSS-treated group was higher than that in the female WT AOM/DSS-treated group (**Figure 7E**).

### Correlation Between the Gut Microbiota and Tumorigenesis Index

LEfSe revealed the differential effect of Nrf2 KO on the abundance of *L. murinus* and *B. vulgatus* depending on sex and CRC induction, respectively.

**Figure 8:**
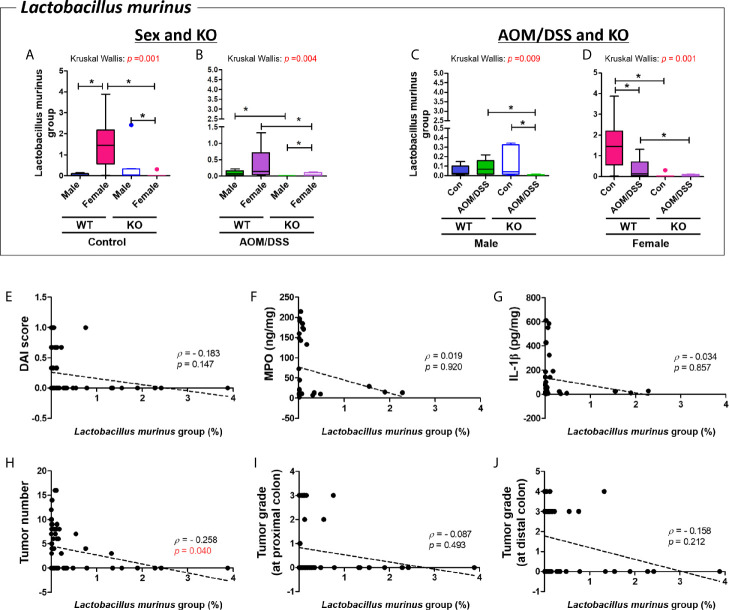
Negative correlation between *Lactobacillus murinus* and tumor numbers in the colon. **(A–D)** Abundance (%) of *Lactobacillus murinus* in the groups with “Sex and KO” criteria **(A, B)**, as well as with “AOM/DSS and KO” criteria **(C, D)**. Data are expressed as the mean ± SEM. Whiskers show the minimum and maximum values. The *p*-values calculated using the Kruskal–Wallis test are shown in the figure; *p < 0.05, comparison between two groups (Mann–Whitney U-test). CRC, colorectal cancer; WT, wild-type; Nrf2 KO, Nrf2 knockout; Con, control; AOM, azoxymethane; DSS, dextran sodium sulfate. **(E)** DAI score, **(F)** MPO concentration, **(G)** IL-1β concentration, **(H)** total tumor numbers, **(I)** grade of tumor arising from the proximal colon, and **(J)** grade of tumor arising from the distal colon. The abundance of *Lactobacillus murinus* was significantly correlated with total tumor numbers **(H)**. *p* < 0.05 (Spearman’s correlation test) **(E–J)**. Dotted line indicates the regression line. The correlations were analyzed using the Spearman’s rank correlation coefficient method (also known as Spearman’s rho) and linear regression. DAI, disease activity index; MPO, myeloperoxidase. Tumor grade: 0, no tumor; 1, low-grade adenoma; 2, high-grade adenoma; 3, mucosal invasive adenocarcinoma; 4, submucosal invasive adenocarcinoma.

The abundance of *L. murinus* in the female WT control group was higher than that in the male WT control group. However, sex-specific changes in the abundance of *L. murinus* were not observed in the Nrf2 KO group (*p* = 0.003 for male WT control group vs. female WT control group and *p* = 0.001 for female WT control group *vs*. female Nrf2 KO control group) (**Figures 8A, D**). The abundance of *L. murinus* in the male Nrf2 KO control group was higher than that in the female Nrf2 KO control group (*p* = 0.050) (**Figure 8A**). Sex-specific changes in the abundance of *L. murinus* were observed in the Nrf2 KO AOM/DSS-treated groups (*p* = 0.049 for male Nrf2 KO AOM/DSS-treated *vs*. female Nrf2 KO AOM/DSS-treated) but not in the WT AOM/DSS-treated groups (**Figure 8B**). However, the abundance of *L. murinus* in the AOM/DSS-treated group was lower than that in the control group (*p* = 0.021 for male Nrf2 KO control *vs*. male Nrf2 KO AOM/DSS-treated and *p* = 0.021 for female WT control *vs*. female WT AOM/DSS-treated) (**Figures 8C, D**). Next, the effects of *L. murinus* on tumorigenesis were evaluated based on the levels of inflammatory markers, the number of tumors, and tumor grade (**Figures 8E–J**). The abundance of *L. murinus* was significantly and negatively correlated with the number of tumors in the whole colon (Spearman’s rho = −0.258; *p* = 0.040) (**Figure 8H**).

**Figure 9:**
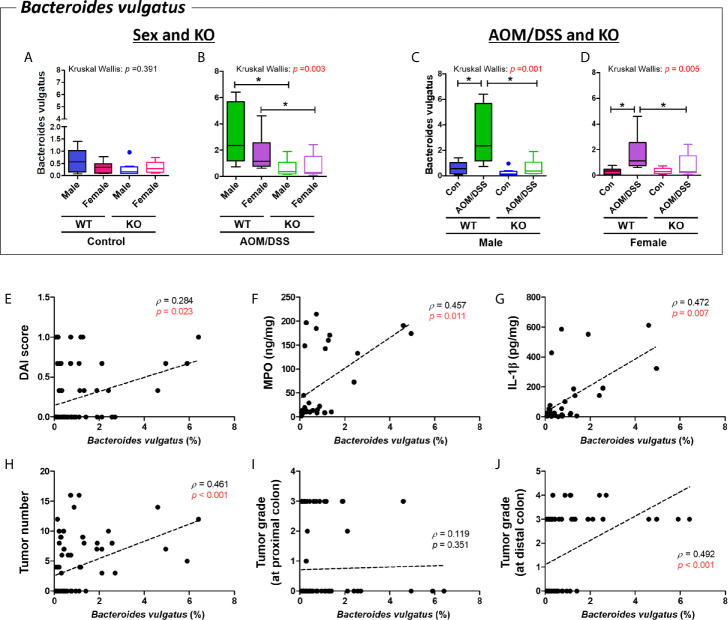
Positive correlation between *Bacteroides vulgatus* and colitis-associated CRC indices. **(A–D)** Abundance (%) of *Bacteroides vulgatus* in the groups with “Sex and KO” criteria **(A, B)**, as well as with “AOM/DSS and KO” criteria **(C, D)**. Data are expressed as the mean ± SEM. Whiskers show the minimum and maximum values. The *p*-values calculated from the Kruskal–Wallis test are shown in the figure; *p < 0.05, comparison between two groups (Mann–Whitney U-test). CRC, colorectal cancer; WT, wild-type; KO, Nrf2 knockout; Con, control; AOM, azoxymethane; DSS, dextran sodium sulfate; SEM, standard error of mean. **(E-J)** The abundance of *Bacteroides vulgatus* was significantly correlated with tumor indices. **(E)** DAI score, **(F)** MPO concentration, **(G)** IL-1β concentration, **(H)** total tumor numbers, **(I)** grade of tumor arising from the proximal colon, and **(J)** grade of tumor arising from the distal colon. *p* < 0.05 obtained from Spearman’s correlation analysis **(E–J)**. Dotted line indicates the regression line. The correlations were analyzed using Spearman’s rank correlation coefficient (also known as Spearman’s rho) and linear regression. DAI, disease activity index; MPO, myeloperoxidase. Tumor grade: 0, no tumor; 1, low-grade adenoma; 2, high-grade adenoma; 3, mucosal invasive adenocarcinoma; 4, submucosal invasive adenocarcinoma.

*B. vulgatus* was enriched in the WT AOM/DSS-treated groups but not in the Nrf2 KO AOM/DSS-treated groups (*p* = 0.006 for male WT control *vs*. male WT AOM/DSS-treated and *p* = 0.002 for female WT control *vs*. female AOM/DSS-treated) (**Figures 9C, D**). The abundance of *B. vulgatus* varied depending on sex (**Figures 9A, B**). Interestingly, the abundance of *B. vulgatus* in the Nrf2 KO groups was lower than that in the WT groups (*p* = 0.005 for male WT AOM/DSS-treated vs. male Nrf2 KO AOM/DSS-treated and *p* = 0.027 for female WT AOM/DSS-treated *vs*. female Nrf2 KO AOM/DSS-treated) (**Figures 9B, D**). Next, the correlation between the abundance of *B. vulgatus* and tumorigenesis was evaluated based on the levels of inflammatory markers, the number of tumors, and tumor grade. The abundance of *B. vulgatus* was positively correlated with the inflammatory markers, including the DAI score, levels of MPO and IL-1β in the colonic mucosa, tumor numbers, and high-grade adenoma, especially, developed mucosal and submucosal invasive adenocarcinoma at the distal part of the colon (DAI score, Spearman’s rho = 0.284 and *p* = 0.023; MPO, Spearman’s rho = 0.457 and *p* = 0.011; IL-1β, Spearman’s rho = 0.472 and *p* = 0.007; tumor numbers in the whole colon, Spearman’s rho = 0.461 and *p* < 0.001; high-grade tumors at the distal part of the colon, Spearman’s rho = 0.492 and *p* < 0.001) (**Figures 9E–J**).

### Enterotypes Constituting the Gut Microbiota

To classify the properties of intestinal microflora that cannot be determined using LEfSe, all samples were further assessed based on the enterotypes. The optimal number “k” was determined based on the highest value of the CH index (**Supplementary Figure S6**). All groups were divided into two enterotypes because the highest CH value was 2.

Enterotype 1 (E1) of the control group with “Sex and KO” criteria comprised the gut microbiota of eight male WT control, two female WT control, seven male Nrf2 KO control, and five female Nrf2 KO control mice. The predominant genus in E1 of the control group was *Muribaculum* (17%), followed by KE159538_g (8%) and PAC000186_g (7%) (**Supplementary Figure S7C**). In contrast, enterotype 2 (E2) of the control group with “Sex and KO” criteria comprised the gut microbiota of zero male WT control, six female WT control, one male Nrf2 KO control, and five female Nrf2 KO control mice. The predominant genus in E2 of the control group was *Akkermansia* (39%), followed by *Muribaculum* (10%) and PAC000186_g (9%) (**Supplementary Figure S7C**). E1 of the AOM/DSS-treated group with “Sex and KO” criteria comprised the gut microbiota of four male WT AOM/DSS-treated, four female WT AOM/DSS-treated, seven male Nrf2 KO AOM/DSS-treated, and three female Nrf2 KO AOM/DSS-treated mice. The predominant genus in E1 of the AOM/DSS-treated group was *Akkermansia* (46%), followed by *Muribaculum* (12%) and PAC000186_g (7%) (**Supplementary Figure S7D**). E2 of the AOM/DSS-treated group with “Sex and KO” criteria comprised the gut microbiota of four male WT AOM/DSS-treated, four female WT AOM/DSS-treated, one male Nrf2 KO AOM/DSS-treated, and five female Nrf2 KO AOM/DSS-treated mice. The predominant genus in E2 of the AOM/DSS-treated group was *Muribaculum* (14%), followed by *Prevotella* (13%) and PAC000186_g (10%) (**Supplementary Figure S7D**). At the species level, E1 (dominant males) and E2 (dominant females) of the control group predominantly comprised unclassified species (9%) and *A. muciniphila* (39%), respectively (**Supplementary Figure S7A**). Meanwhile, *A. muciniphila* (46%) and PAC002481_s (13%) were the predominant species in E1 (dominant Nrf2 KO males) and E2 of the AOM/DSS-treated group, respectively (**Supplementary Figure S7B**).

E1 of the male group with “AOM/DSS and KO” criteria comprised the gut microbiota of eight WT control, four WT AOM/DSS-treated, seven Nrf2 KO control, and one Nrf2 KO AOM/DSS-treated mice. The predominant genus in E1 of the male group was *Muribaculum* (18%), followed by *Prevotella* (11%) and *Muribaculaceae*_uc (7%) (**Supplementary Figure S7G**). E2 of the male group with “AOM/DSS and KO” criteria comprised the gut microbiota of zero WT control, four WT AOM/DSS-treated, one Nrf2 KO control, and seven Nrf2 KO AOM/DSS-treated mice. The predominant genus in E2 of the male group was *Akkermansia* (45%), followed by *Muribaculum* (10%) and PAC000186_g (8%) (**Supplementary Figure S7G**). E1 of the female group with “AOM/DSS and KO” criteria comprised the gut microbiota of two WT control, five WT AOM/DSS-treated, four Nrf2 KO control, and five Nrf2 KO AOM/DSS-treated mice. The predominant genus in E1 of the female group was *Muribaculum* (15%), followed by PAC000186_g (12%) and KE159538_g (10%) (**Supplementary Figure S7H**). E2 of the female group with “AOM/DSS and KO” criteria comprised the gut microbiota of six WT control, three WT AOM/DSS-treated, four Nrf2 KO control, and three Nrf2 KO AOM/DSS-treated mice. The predominant genus in E2 of the female group was *Akkermansia* (46%), followed by *Muribaculum* (10%) and PAC000186_g (7%) (**Supplementary Figure S7H**). At the species level, PAC002481_s (11%) and *A. muciniphila* (45%) were the predominant species in E1 (dominant control groups) and E2 of the male groups (dominant Nrf2 KO male AOM/DSS group), respectively (**Supplementary Figure S7E**). Furthermore, PAC001064_s (9%) and *A. muciniphila* (46%) were the predominant species in the E1 (dominant AOM/DSS groups) and E2 of the female groups (dominant WT female control), respectively (**Supplementary Figure S7F**). These results indicate that *A. muciniphila* was the predominant species in female control mice, as well as in male AOM/DSS-treated and Nrf2 KO mice.

## Discussion

**Figure 10:**
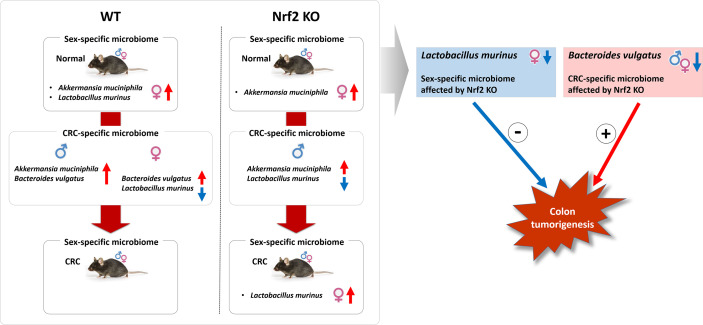
Illustrative summary. The findings of this study indicated that Nrf2 KO was associated with sex-specific and CRC-specific alterations in the gut microbiome composition in the mouse model. Nrf2 KO differentially altered the abundance of two bacterial species, which are *L. murinus* and *B*. *vulgatus*, depending on sex and CRC induction. Interestingly, the sex-specific difference in the abundance of *L. murinus*, which was modulated by Nrf2 KO, was negatively correlated with the colitis-associated CRC index. In contrast, the CRC-specific difference in the abundance of *B*. *vulgatus*, which was also regulated by Nrf2 KO, was positively correlated with colitis-associated CRC indices. WT, wild-type; KO, knockout; CRC, colorectal cancer; +, positive correlation; -, negative correlation.

The findings of this study indicated that Nrf2 KO was associated with sex-specific and CRC-specific alterations in the gut microbiome composition in the mouse model. Nrf2 KO differentially altered the abundance of two bacterial species, which are *L. murinus* and *B. vulgatus*, depending on sex and CRC induction. Interestingly, the sex-specific difference in the abundance of *L. murinus*, which was modulated by Nrf2 KO, was negatively correlated with the colitis-associated CRC index. In contrast, the CRC-specific difference in the abundance of *B. vulgatus*, which was also regulated by Nrf2 KO, was positively correlated with colitis-associated CRC indices (**Figure 10**).

The balance of the gut microbiome is critical for the maintenance of host homeostasis. Dysbiosis can lead to the development of diseases, such as cancer, metabolic diseases, allergies, and immunological disorders (Shanahan, 2013). Previously, we investigated the role of Nrf2 in colitis-associated CRC progression. The Nrf2 KO transgenic mice were treated with AOM/DSS (Song et al., 2020a; Song et al., 2021) which is a widely used model to examine the molecular pathogenesis of colitis-associated CRC (Suzuki et al., 2004; Thaker et al., 2012). Previously, we reported that 17β-estradiol markedly inhibited the AOM/DSS-induced adenoma/cancer incidence rate at the distal part of the colon in male Nrf2 KO mice by downregulating the NF-κB-related signaling pathway and upregulating ERβ protein expression (Song et al., 2020a). Interestingly, the expression levels of NF-κB-mediated proinflammatory mediators in the Nrf2 KO group were lower than those in the WT group irrespective of 17β-estradiol treatment (Song et al., 2020a). The previously reported results of the stool samples were used in this study to determine the correlation between the host gut microbiome composition and other factors, such as sex, CRC, and Nrf2 KO.

Various indicators of gut microbial diversity have been developed to estimate and examine the characteristics of microbial communities. Generally, alpha diversity, an indicator of species richness and diversity, is a characteristic of the gut microbiota of healthy individuals (Le Chatelier et al., 2013). In contrast, low diversity of the gut microbiome is associated with various inflammatory and metabolic diseases, such as obesity (Turnbaugh et al., 2009), inflammatory bowel disease (Ott and Schreiber, 2006), and CRC (Ai et al., 2019). Previous studies have reported that the gut microbiota of DSS-treated C57BL/6 mice exhibited decreased microbial diversity (Hakansson et al., 2015). In the physiological conditions, females exhibited a higher α-diversity than males in humans (Kim et al., 2020) and BALB/c and B6 mouse strains (Elderman et al., 2018). Furthermore, an independent analysis of the gut microbiota using 89 different inbred mouse strains revealed that the gut microbiota composition and diversity varied between male and female mice within each strain (Org et al., 2016). In particular, the sex-specific differences in gut microbiota composition were observed in the C57BL/6J and C3H/HeJ strains (Org et al., 2016). However, most preclinical studies have used male animals, whereas some studies have not specified the sex of the animal (Jahng and Kim, 2017). In this study, both male and female mice were used to investigate the effect of sex, CRC, and Nrf2 on the gut microbiota composition and diversity. The α-diversity indices (ACE, Chao1, Jackknife, Shannon, and Simpson indices) of the gut microbiome in the female WT control group were significantly lower than those in the male WT control group. However, the sex-specific differences in the gut microbiome α-diversity were not observed in the Nrf2 KO and/or AOM/DSS-treated groups. The α-diversity decreased upon treatment with AOM/DSS only in males (in both WT and Nrf2 KO mice) but not in females. These results were not consistent with those of previous studies that reported sex-specific differences in gut microbial diversity. However, the results of this study are consistent with those of our previous study involving an identical C57BL/6 background mouse strain (Son et al., 2019a). Similarly, there was a distinct sex differences on microbiota in the control group which also disappeared in the IL-10 KO-induced inflammatory bowel disease model. Furthermore, sex-specific alterations in the gut microbial diversity were not observed in our previous studies using F344 rats and ICR mice (Lee et al., 2018; Song et al., 2020b). Thus, there are inconsistent findings on the sex-specific alterations in gut microbial composition. Each weighted index measures different aspects of diversity. Hence, the diversity index does not reveal the actual diversity. Furthermore, these disparities might be due to differences in animal models, housing conditions, and diet used in different studies.

The phyla *Firmicutes* and *Bacteroidetes* are reported to be the predominant bacterial populations in the human gastrointestinal microbiome (Collado et al., 2012; Breban, 2016). The F/B ratio is used as a representative index to compare different microbial communities (Mariat et al., 2009). A low F/B ratio is reported to be associated with a healthy condition (Turnbaugh et al., 2006; Mariat et al., 2009) whereas a high F/B ratio is associated with obesity (Raskov et al., 2017) and CRC (Gagniere et al., 2016; Lucas et al., 2017). This indicates that the F/B ratio is a key indicator of gut dysbiosis. Epidemiological studies have indicated that the F/B ratio is associated with obesity and an increased risk of CRC in 30%–70% of patients (Kim et al., 2020). One study reported that men exhibited a higher F/B ratio at a body mass index of 33 and a lower F/B ratio at a body mass index > 33 when compared with women (Haro et al., 2016). However, under physiological conditions, the F/B ratio in women is higher than that in men. In this study, the F/B ratio did not vary with sex, AOM/DSS treatment, or Nrf2 KO. These results are consistent with those of our previous study involving ICR-background mice (Song et al., 2020b). The F/B ratio was not affected by sex, OVX-mediated endogenous estrogen depletion, and AOM/DSS treatment in ICR mice (Song et al., 2020b). However, the F/B ratio significantly decreased in the male AOM/DSS-treated group upon treatment with 17β-estradiol (Song et al., 2020b). Another study reported that BALB/c and B6 mouse strains did not exhibit sex-specific alteration in the F/B ratio (Elderman et al., 2018). Previous studies have reported that the abundance of *Firmicutes* in patients with inflammatory bowel disease was lower than that in healthy controls, which indicated a low F/B ratio (Gophna et al., 2006; Frank et al., 2007). Although the F/B ratio could be a useful indicator of disease progression, various implications of the F/B ratio have been reported by different studies. Therefore, the interpretation of the F/B ratio is controversial.

In this study, we identified sex-specific (*A. muciniphila* and *L. murinus*), CRC-specific (*A. muciniphila*, *B. vulgatus*, *L. murinus*, and *M. intestinale*), and Nrf2 KO-specific (*B. vulgatus*, *L. murinus*, and *M. intestinale*) taxonomic biomarkers. Of these, Nrf2 KO affected the abundance of only *L. murinus* (a sex-specific commensal bacterium) and *B. vulgatus* (a CRC-specific opportunistic pathogen). Interestingly, the abundance of *A. muciniphila*, which was both a sex-specific and a CRC-specific biomarker, was not affected by Nrf2 KO. *A. muciniphila*, a mucin-degrading bacterium, can convert mucin to short-chain fatty acids, such as acetic acid and propionic acid (Huang et al., 2015; Ottman et al., 2016). A large cohort study demonstrated that women in the Netherlands exhibited an increased abundance of *A. muciniphila* even after correcting for all diverse factors, including diet, lifestyle, and medication for maintaining health (Sinha et al., 2019). In the Japanese population, the abundance of members belonging to the genus *Akkermansia* in women was significantly higher than that in men (Gao et al., 2018). In C57BL/6 mice, the abundance of *A. muciniphila* in females is higher than that in males (Kaliannan et al., 2018), which is consistent with the results of this study. *A. muciniphila*, a commensal bacterium, could be involved in the proinflammatory pathways and the activation of chemotaxis and the complement cascade (Derrien et al., 2011). Furthermore, *A. muciniphila* is reported to be a promising probiotic (Zhang et al., 2019b). However, there are conflicting reports on the role of *A. muciniphila* as commensal and opportunistic bacteria. *A. muciniphila* functions as an opportunistic pathogen to exacerbate intestinal inflammation in mice infected with *Salmonella typhimurium* by dysregulating host homeostasis in the mucus layer (Ganesh et al., 2013). Furthermore, patients with multiple sclerosis exhibited a higher abundance of *A. muciniphila* than healthy controls (Cekanaviciute et al., 2017) or a monozygotic twin pair without multiple sclerosis (Berer et al., 2017). Therefore, this study did not classify *A. muciniphila* as a commensal or opportunistic bacterium but classified it as a dual-role bacterium with different roles depending on the conditions. Most recent studies focus on the correlation between *A. muciniphila* and diseases, and there are limited studies on the causal relationship between the abundance of *A. muciniphila* and diseases.

Generally, *L. murinus* is the most frequently detected *Lactobacillus* strain. *Lactobacillus* species are predominantly enriched in the vaginal tracts of European women (Fettweis et al., 2014) and female mice (Fraga et al., 2005). Fraga et al. reported that an indigenous *L. murinus* strain LbO2 isolated from the vaginal tract of a female mouse exhibited probiotic properties, including characteristics, such as acid and bile salt tolerance, survival in the urinary tract, attachment ability to uroepithelial cells, and antimicrobial activity (Fraga et al., 2005). Recently, Singer et al. reported that *L. murinus* strain V10 protected against neonatal dysbiosis and late-onset sepsis (Singer et al., 2019). The administration of *L. murinus* strain markedly improved the bioavailability of glycyrrhizic acid, which is the major bioactive triterpene glycoside with anti-inflammatory, antioxidation, antiviral, and hepatoprotective properties, under pathological conditions in a rat model (Yuan et al., 2020). In this study, the abundance of *L. murinus* in the female WT control group was higher than that in the male WT control group. However, the abundance of *L. murinus* in the female WT AOM/DSS-treated group was lower than that in the female WT control group. Furthermore, correlation analysis between the gut microbiome and disease index revealed that the abundance of *L. murinus* was negatively correlated with the number of colon tumors. Previously, we reported the lack of sex-specific differences in the abundance of *L. murinus*. However, the abundance of *L. murinus* group in the male 17β-estradiol-supplemented and AOM/DSS-treated group was higher than that in the male AOM/DSS-treated group (Song et al., 2020b). Moreover, *L. plantarum* FC225, which was isolated from fermented cabbages, and *L. reuteri* exhibit antioxidant activity through the Nrf2 signaling pathway in hyperlipidemic mice (Gao et al., 2013) and chemotherapy-induced oral mucositis mice (Gupta et al., 2020), respectively. However, there are no studies on the correlation between *L. murinus* and Nrf2, especially the procarcinogenic effects of Nrf2.

*B. vulgatus* is the predominant species in the human colon microbiota (McCarthy et al., 1988). Bacteroides species are reported to induce colitis based on the host genotype in a mouse model of inflammatory bowel disease (Bloom et al., 2011). The abundance of *B. vulgatus* is high in patients with Crohn’s disease (Ruseler-van Embden et al., 1989). Bloom et al. reported that *B. vulgatus* and *B. thetaiotaomicron* induced severe ulcerative disease (Bloom et al., 2011). Furthermore, metagenome sequencing revealed that *B. vulgatus* and *Eubacterium rectale* exhibited the highest abundance in the feces of patients with mild and moderate non-alcoholic fatty liver disease, while *B. vulgatus* and *E. coli* were the most abundant in patients with liver fibrosis (Loomba et al., 2017). Consistent with previous reports, this study demonstrated that the abundance of *B. vulgatus* in male and female AOM/DSS-treated mice was higher than that in the male and female control mice. Interestingly, the abundance of *B. vulgatus* in the Nrf2 KO AOM/DSS-treated group was similar to that in the WT AOM/DSS-treated group. Furthermore, the abundance of *B. vulgatus* was positively correlated with inflammatory markers (DAI score, and levels of MPO and IL-1β), developed tumor numbers, and high-grade adenoma developed mucosal and submucosal invasive adenocarcinoma, especially, at the distal part of the colon. These results suggest that the abundance of *B. vulgatus*, which acts as an opportunistic pathogen, is positively correlated with procarcinogenic Nrf2 and that Nrf2 may be a useful target to reduce the level of *B. vulgatus* in patients with CRC. However, it is well known that single bacteria do not suffice to explain benefit or pathogenicity. Bacterial interaction can make a big difference (Christofi et al., 2019). However, we could not evaluate the bacterial interaction deeply, and it is the limitation of this study. Similar limitation of this study is that the findings cannot explain the cause-and-effect relationship between gut microbial alteration and host responses in AOM/DSS-induced CRC development. This is because the present study focused only on endpoint comparisons rather than longitudinal studies. To overcome the limitation, pinpointing novel factors that drive CRC through longitudinally-changing holo’omes, which is defined as the combination of host and microbiota genomes, transcriptomes, proteomes, and metabolomes, might be necessary to improve CRC prognosis, diagnosis and therapy (Panagi et al., 2019). Furthermore, in this study, only tumor-bearing mice were evaluated, which tends to be biased toward evaluating the outcome of tumorigenesis rather than the potential cause. However, the correlation analysis in this study suggests that the sex-specific alteration in the abundance of *L. murinus* and the CRC-specific alteration in the abundance of *B. vulgatus*, which are both affected by Nrf2 KO, are negatively and positively correlated with colitis-associated CRC indices, respectively. To verify the effect of the gut microbiome on host responses, future studies must supplement *L. murinus* or *B. vulgatus* to the AOM/DSS-induced CRC mouse models. It is necessary to determine how *B. vulgatus* or *L. murinus* changes when inflammatory molecules are blocked or supplemented.

In conclusion, the findings of this study suggest that Nrf2 can differentially modulate the gut microbiota composition depending on sex and CRC. Thus, Nrf2, which is regulated by estrogen, is an important factor that can explain the sex-specific difference in the risk of developing CRC.

## Data Availability Statement

The datasets generated for this study are available in NCBI BioProject, under accession number PRJNA674731, https://www.ncbi.nlm.nih.gov/bioproject/PRJNA674731.

## Ethics Statement

The experimental procedures were approved by the Institutional Animal Care and Use Committee of the Seoul National University Bundang Hospital (approval number: BA1705-223/043-01).

## Author Contributions

C-HS analyzed the results and written the manuscript. NK designed the study and supervised the writing of the manuscript. RN, SC, JY and HN conducted the animal experiments. Y-JS reviewed the manuscript critically. All authors contributed to the article and approved the submitted version.

## Funding

This work was supported by a grant from the National Research Foundation of Korea (NRF) funded by the government of the Republic of Korea (2019R1A2C2085149).

## Conflict of Interest

The authors declare that the research was conducted in the absence of any commercial or financial relationships that could be construed as a potential conflict of interest.
