# Supplementary material for: Changes in Microbial Community Composition Related to Sex and Colon Cancer by Nrf2 Knockout
Source: Front Cell Infect Microbiol. 2021 Jun 23;11:636808. doi: 10.3389/fcimb.2021.636808 (PMC8261249; doi:10.3389/fcimb.2021.636808)
Supplement: Supplementary file 1 [file Table_1.docx]

Supplementary Material

Changes in Microbial Community Composition Related to Sex and Colon Cancer by Nrf2 Knockout

Chin-Hee Song, Nayoung Kim^*^, Ryoung Hee Nam, Soo In Choi, Jeong Eun Yu, Heewon Nho, and Young-Joon Surh

*** Correspondence:** Nayoung Kim: nakim49@snu.ac.kr


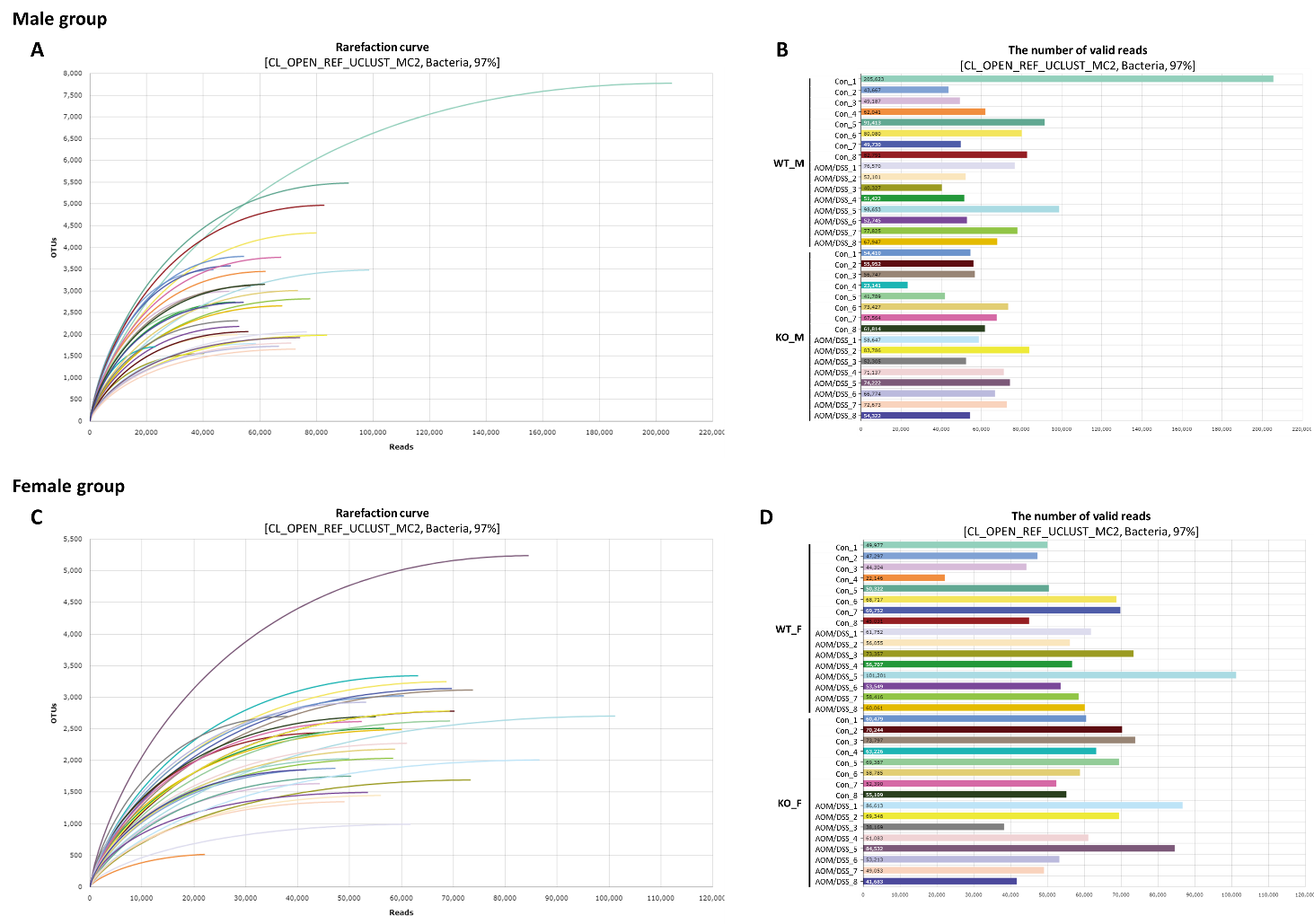


**Supplementary Figure S1.** Rarefaction curves and the number of valid reads of the 97% OTUs observed species. (A-D) Rarefaction curves (A,C) and the number of valid reads (B,D) in males including WT male control, WT male AOM/DSS, Nrf2 KO male control, and Nrf2 KO male AOM/DSS (A,B) and female groups including WT female control, WT female AOM/DSS, Nrf2 KO female control, and Nrf2 KO female AOM/DSS (C,D).
