# Supplementary material for: Changes in Microbial Community Composition Related to Sex and Colon Cancer by Nrf2 Knockout
Source: Front Cell Infect Microbiol. 2021 Jun 23;11:636808. doi: 10.3389/fcimb.2021.636808 (PMC8261249; doi:10.3389/fcimb.2021.636808)
Supplement: Supplementary file 3 [file Table_3.docx]

Supplementary Material

Changes in Microbial Community Composition Related to Sex and Colon Cancer by Nrf2 Knockout

Chin-Hee Song, Nayoung Kim^*^, Ryoung Hee Nam, Soo In Choi, Jeong Eun Yu, Heewon Nho, and Young-Joon Surh

*** Correspondence:** Nayoung Kim: nakim49@snu.ac.kr


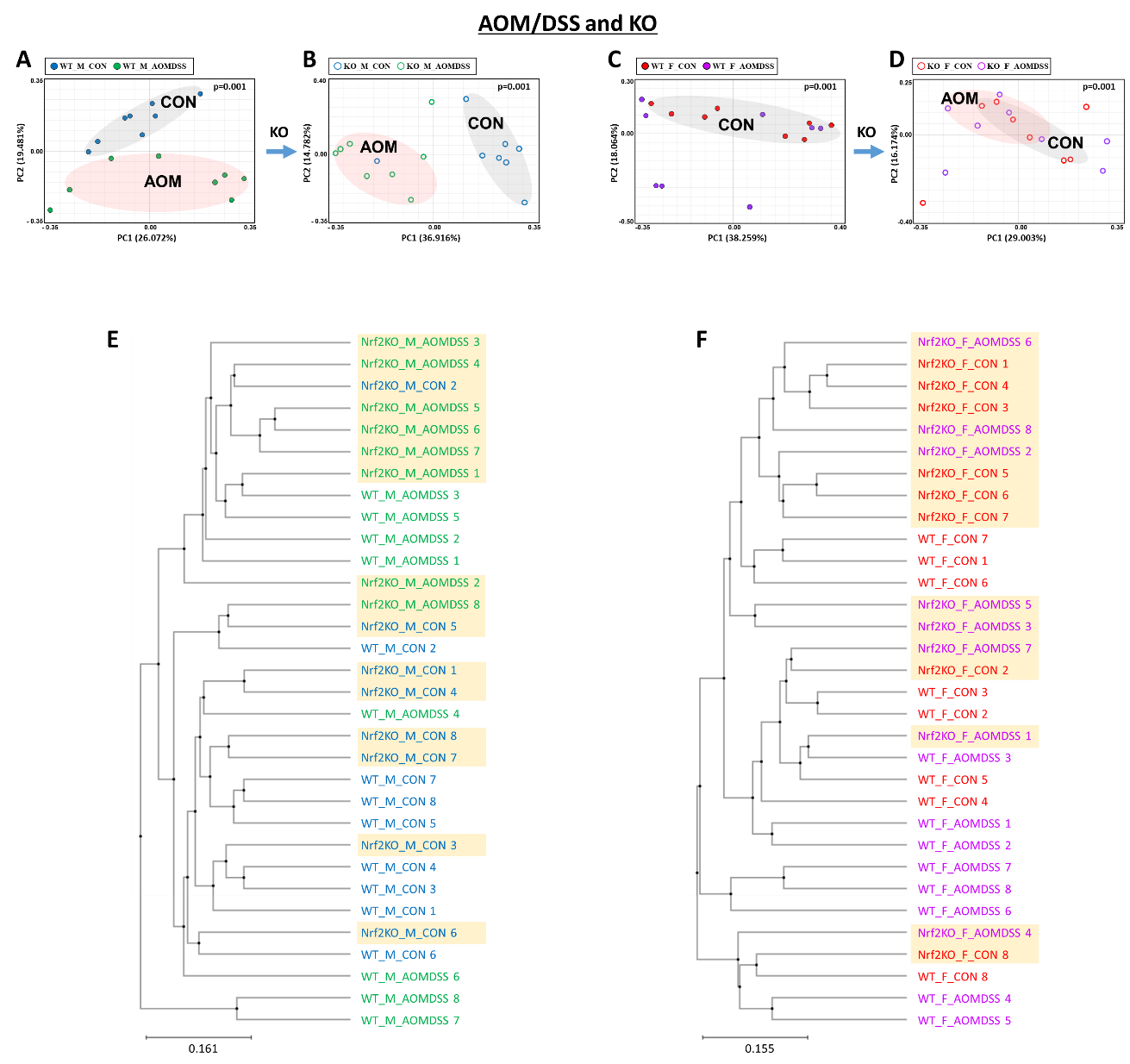


**Supplementary Figure S3.** Beta diversity analysis of gut microbiota by AOM/DSS-induced CRC development. (A-D) UniFrac-based principal coordinate analysis (PCoA) of 16S rRNA sequence from 64 fecal samples. Samples derived from males in WT (A) and Nrf2 KO (B) and from females in WT (C) and Nrf2 KO (D) were clustered by Generalized UniFrac method at the species level. PERMANOVA test for the dissimilarity of bacterial population structures was performed in (A), (B), (C), and (D). (E,F) UniFrac-based unweighted pair-group method with arithmetic mean (UPGMA) tree of 16S rRNA sequence from 64 fecal samples. Males (E) and females (F) including WT controls, WT AOM/DSS, Nrf2 KO controls, and Nrf2 KO AOM/DSS groups. WT, wild-type; KO, Nrf2 knockout; AOM, azoxymethane; DSS, dextran sodium sulfate; M, male; F, female.
