# Supplementary material for: Changes in Microbial Community Composition Related to Sex and Colon Cancer by Nrf2 Knockout
Source: Front Cell Infect Microbiol. 2021 Jun 23;11:636808. doi: 10.3389/fcimb.2021.636808 (PMC8261249; doi:10.3389/fcimb.2021.636808)
Supplement: Supplementary file 4 [file Table_4.docx]

Supplementary Material

Changes in Microbial Community Composition Related to Sex and Colon Cancer by Nrf2 Knockout

Chin-Hee Song, Nayoung Kim^*^, Ryoung Hee Nam, Soo In Choi, Jeong Eun Yu, Heewon Nho, and Young-Joon Surh

*** Correspondence:** Nayoung Kim: nakim49@snu.ac.kr


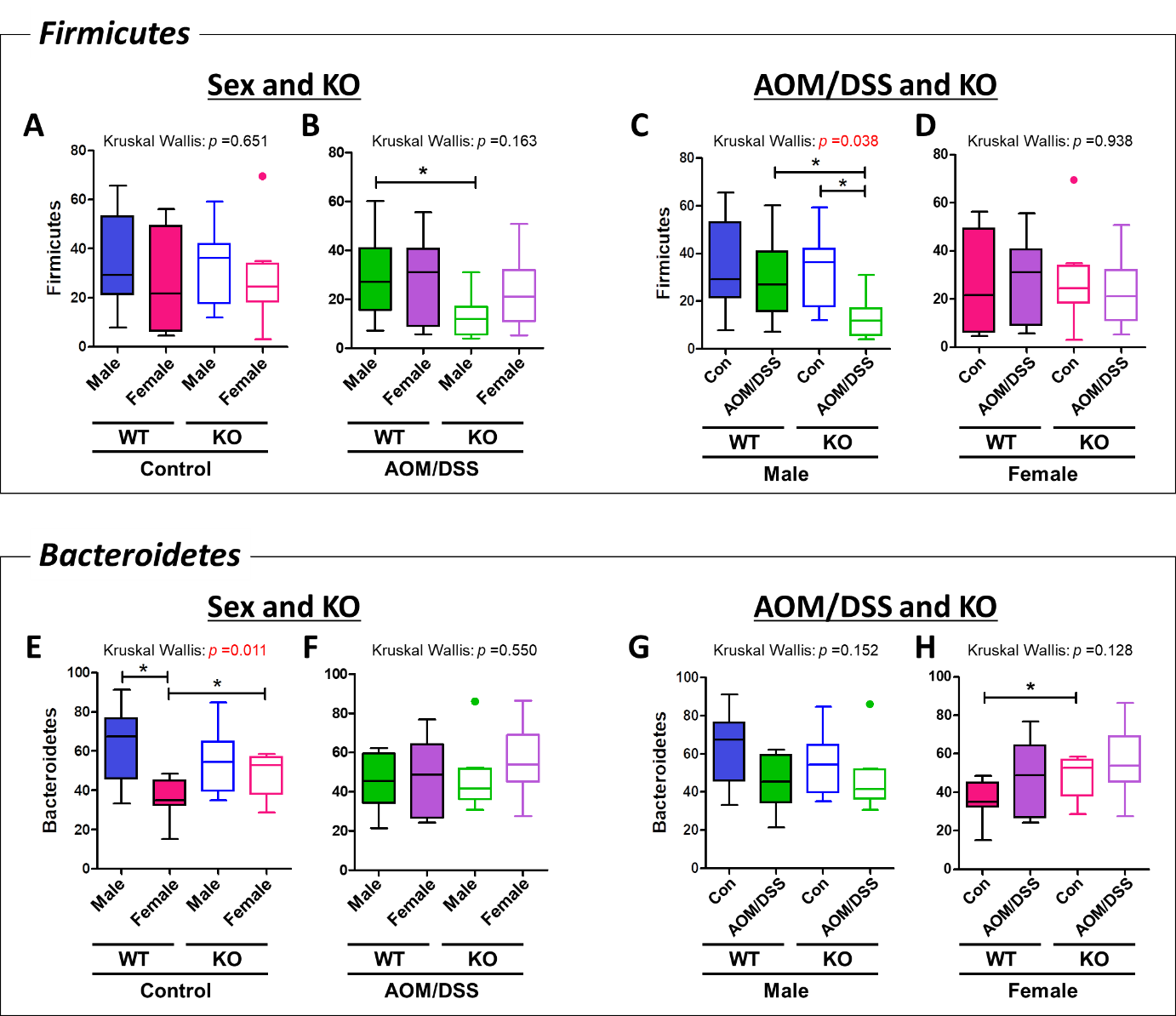


**Supplementary Figure S4.** Gut microbiota composition at the phylum level. (A–H) The abundance of *Firmicutes* (A-D) and *Bacteroidetes* (E-H) in the groups with “Sex and KO” criteria (A, B, E, and F), as well as with “AOM/DSS and KO” criteria (C, D, G, and H). Data are expressed as the mean ± SEM. Whiskers show the minimum and maximum values. The *p*-values calculated from the Kruskal–Wallis test is shown in the figure; ∗*p* < 0.05, comparison between two groups (Mann–Whitney U-test). WT, wild-type; Nrf2 KO, Nrf2 knockout; Con, control; AOM, azoxymethane; DSS, dextran sodium sulfate; SEM, standard error of mean.
