# Supplementary material for: Changes in Microbial Community Composition Related to Sex and Colon Cancer by Nrf2 Knockout
Source: Front Cell Infect Microbiol. 2021 Jun 23;11:636808. doi: 10.3389/fcimb.2021.636808 (PMC8261249; doi:10.3389/fcimb.2021.636808)
Supplement: Supplementary file 5 [file Table_5.docx]

Supplementary Material

Changes in Microbial Community Composition Related to Sex and Colon Cancer by Nrf2 Knockout

Chin-Hee Song, Nayoung Kim^*^, Ryoung Hee Nam, Soo In Choi, Jeong Eun Yu, Heewon Nho, and Young-Joon Surh

*** Correspondence:** Nayoung Kim: nakim49@snu.ac.kr


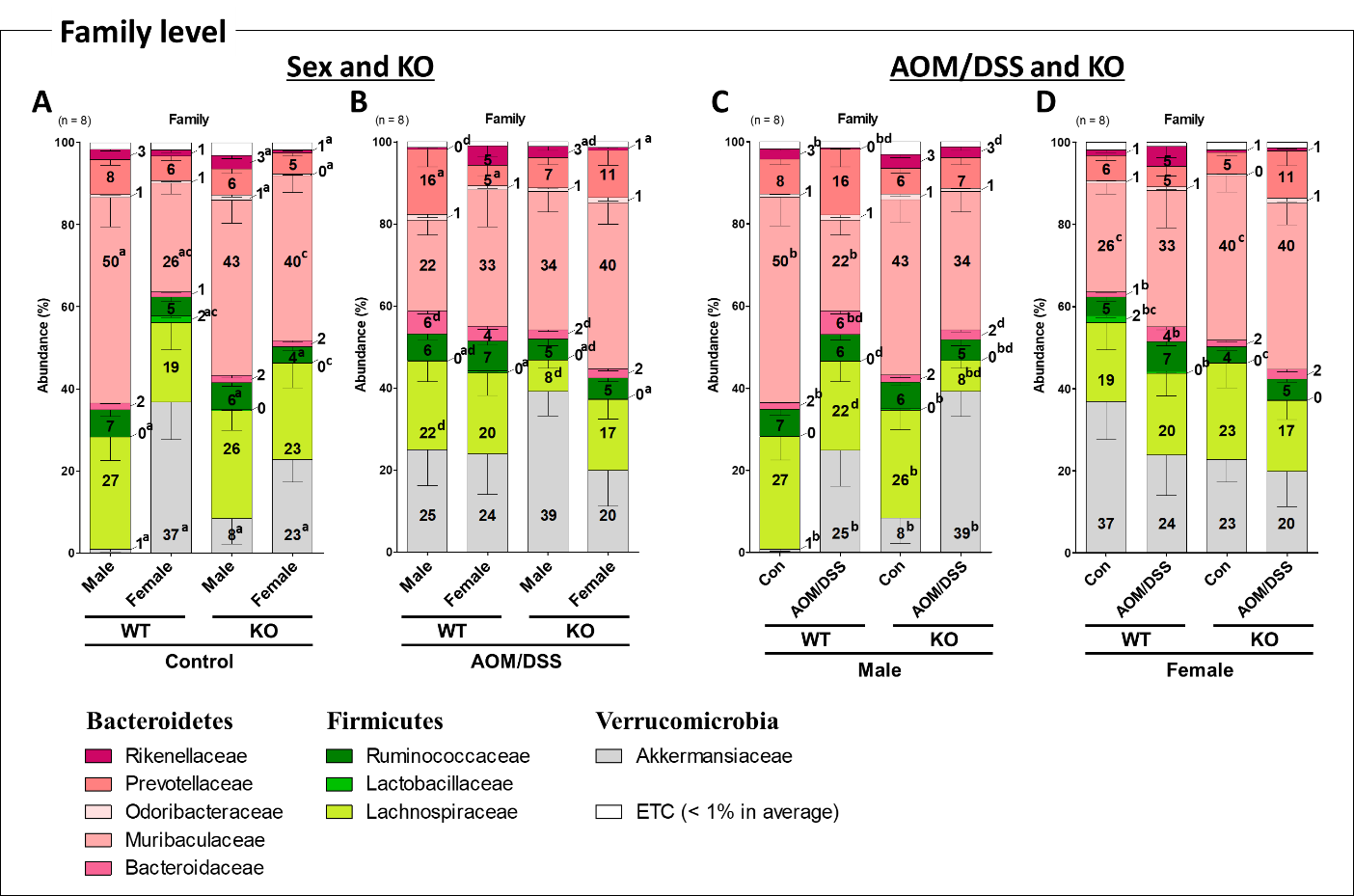


**Supplementary Figure S5.** Gut microbiota compositions at the Family level. (A-D) Taxonomic composition of fecal contests from Sex and KO groups (A,B) and AOM/DSS and KO groups (C,D). The abundance ratio indicates the percentage of each family in total microorganisms. Stacked bars of mean ± SEM. Mann–Whitney U-test for comparison difference between independent two groups was performed in (A), (B), (C), and (D). ^a^, *p* < 0.05 between males vs females; ^b^, *p* < 0.05 between controls vs AOM/DSS groups; ^c^, *p* < 0.05 between WT controls vs Nrf2 KO controls; ^d^, *p* < 0.05 between WT AOM/DSS vs Nrf2 KO AOM/DSS. WT, wild-type; KO, Nrf2 knockout; Con, control; AOM, azoxymethane; DSS, dextran sodium sulfate.
