# Supplementary material for: Changes in Microbial Community Composition Related to Sex and Colon Cancer by Nrf2 Knockout
Source: Front Cell Infect Microbiol. 2021 Jun 23;11:636808. doi: 10.3389/fcimb.2021.636808 (PMC8261249; doi:10.3389/fcimb.2021.636808)
Supplement: Supplementary file 6 [file Table_6.docx]

Supplementary Material

Changes in Microbial Community Composition Related to Sex and Colon Cancer by Nrf2 Knockout

Chin-Hee Song, Nayoung Kim^*^, Ryoung Hee Nam, Soo In Choi, Jeong Eun Yu, Heewon Nho, and Young-Joon Surh

*** Correspondence:** Nayoung Kim: nakim49@snu.ac.kr


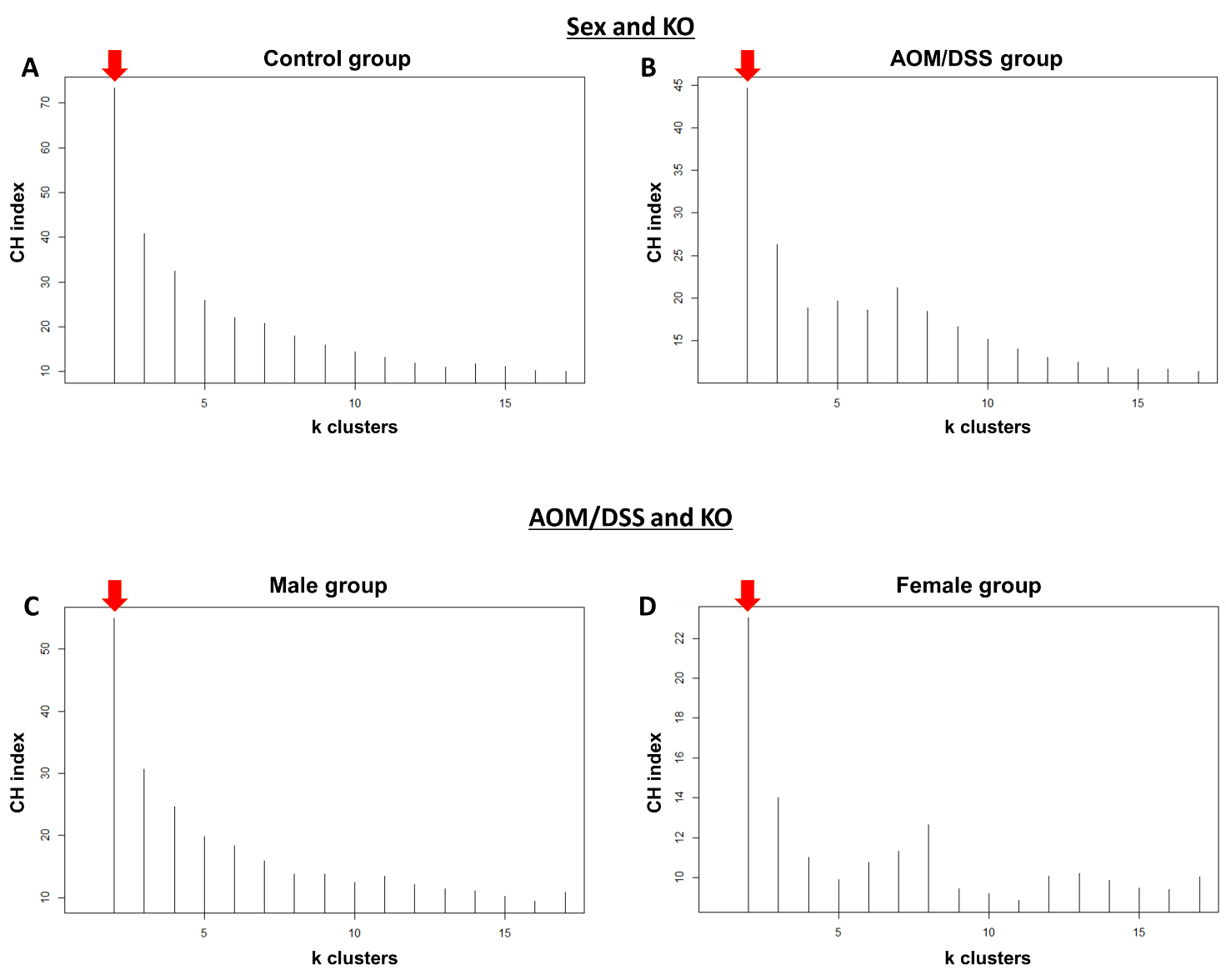


**Supplementary Figure S6.** Calinski-Harabasz (CH) index for the separation of enterotypes. Enterotypes were determined based on the CH index. (A-D) All samples from controls (A) and AOM/DSS group (B) in Sex and KO criteria and from males (C) and females (D) in AOM/DSS and KO criteria were separated into two enterotypes based to the highest CH index when k cluster was 2. KO, Nrf2 knockout; AOM, azoxymethane; DSS, dextran sulfate sodium salt.
