# Supplementary material for: Changes in Microbial Community Composition Related to Sex and Colon Cancer by Nrf2 Knockout
Source: Front Cell Infect Microbiol. 2021 Jun 23;11:636808. doi: 10.3389/fcimb.2021.636808 (PMC8261249; doi:10.3389/fcimb.2021.636808)
Supplement: Supplementary file 7 [file Table_7.docx]

Supplementary Material

Changes in Microbial Community Composition Related to Sex and Colon Cancer by Nrf2 Knockout

Chin-Hee Song, Nayoung Kim^*^, Ryoung Hee Nam, Soo In Choi, Jeong Eun Yu, Heewon Nho, and Young-Joon Surh

*** Correspondence:** Nayoung Kim: [nakim49@snu.ac.kr](mailto:nakim49@snu.ac.kr)


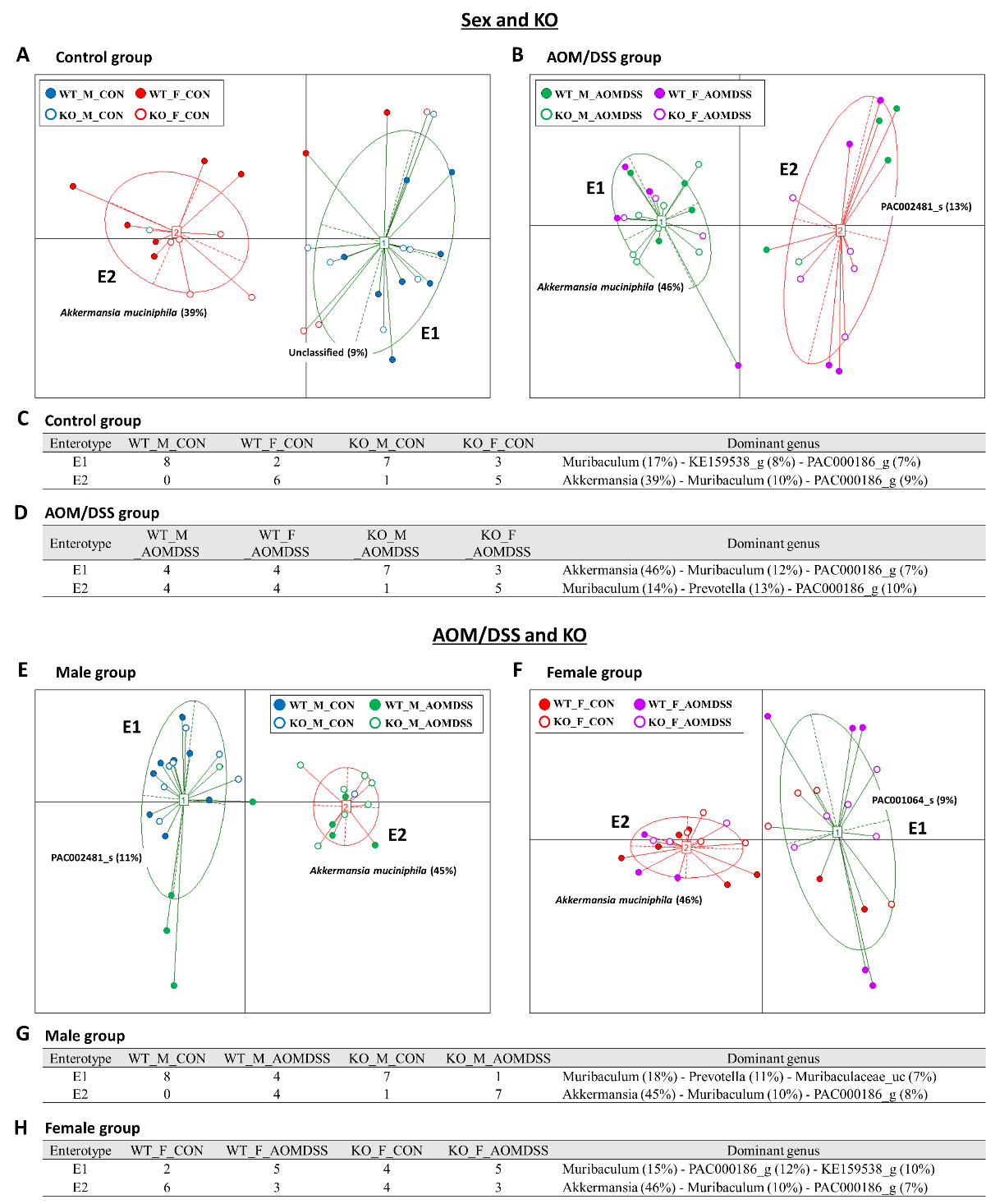


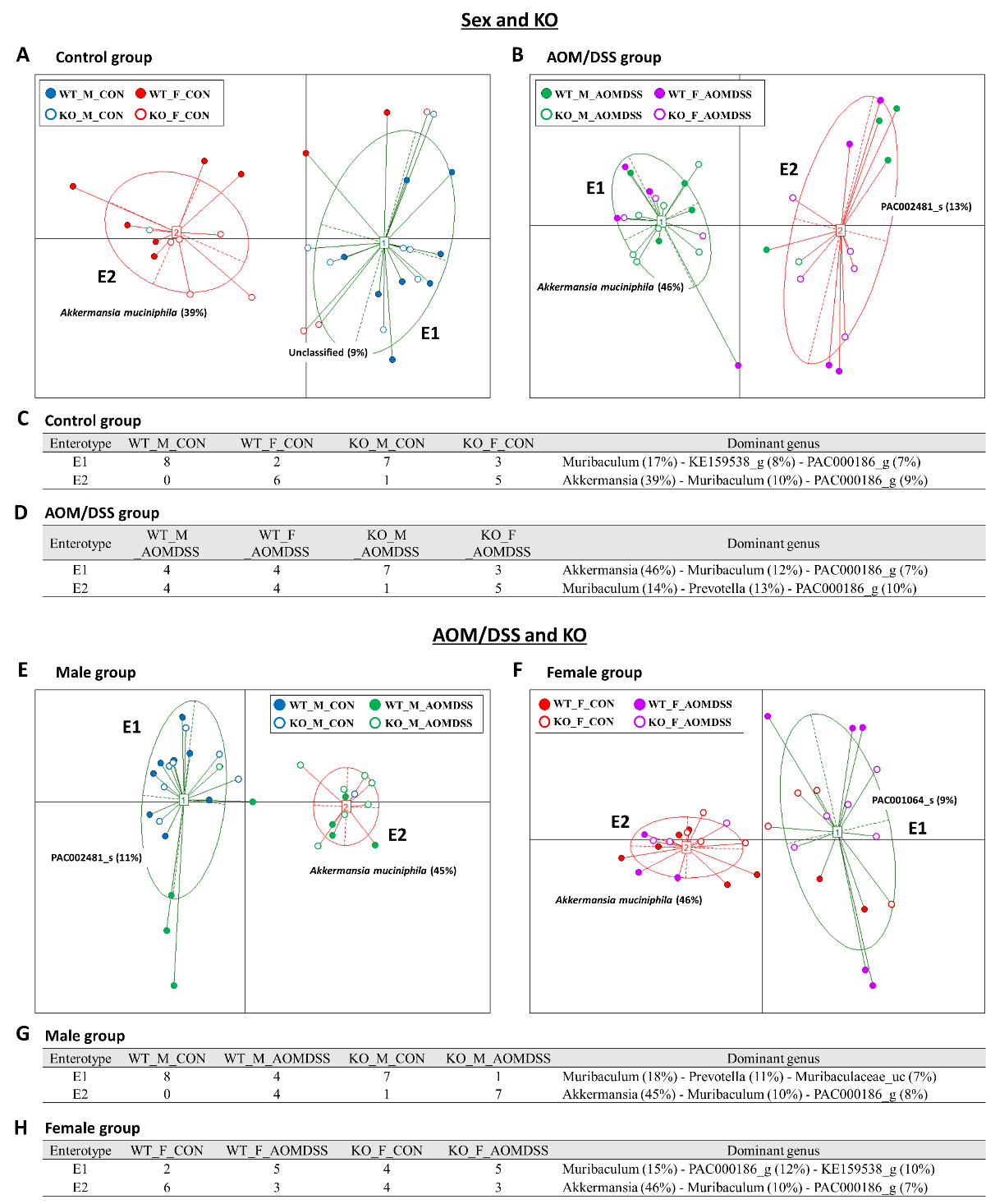


**Supplementary Figure S7.** Enterotype clustering at the species level. (A-D) Two enterotypes (E1 and E2) of control samples (A) and two enterotypes of AOM/DSS group samples (B). The tables show the predominant genus in each enterotype of control (C) and AOM/DSS mice samples (D). (E-H) Two enterotypes of males (E) and two enterotypes of females (F). The tables show the predominant genus in each enterotype of males (G) and female mice samples (H). The optimal cluster number was determined by maximizing the Calinski-Harabasz (CH) index value (**Supplementary Figure S5**). WT, wild-type; KO, Nrf2 knockout; Con, control; AOM, azoxymethane; DSS, dextran sodium sulfate; M, male; F, female.
